# Deep learning based deconvolution methods: A systematic review

**DOI:** 10.1016/j.csbj.2025.05.038

**Published:** 2025-06-11

**Authors:** Alba Lomas Redondo, Jose M. Sánchez Velázquez, Álvaro J. García Tejedor, Víctor Javier Sánchez–Arévalo Lobo

**Affiliations:** a CEIEC, Universidad Francisco de Vitoria (UFV), Pozuelo de Alarcón, 28223, Madrid, Spain; b Grupo de Oncología Molecular, Instituto de Investigaciones Biosanitarias, Facultad de Ciencias Experimentales, Universidad Francisco de Vitoria (UFV), Pozuelo de Alarcón, 28223, Madrid, Spain; c Grupo de Oncología Cutánea, Servicio de Anatomía Patológica, Hospital Universitario 12 de Octubre, Instituto de Investigación Sanitaria Hospital 12 de Octubre (imas12), Avenida de Cordoba s/n, 28041, Madrid, Spain

**Keywords:** Cellular deconvolution, Deep learning, Artificial intelligence, Neural network, RNA–seq, scRNA–seq, Transcriptomics data, Computational biology

## Abstract

Within this systematic review we examine the role of Artificial Intelligence (AI) and Deep Learning (DL) in the development of cellular deconvolution tools, with an special focus on their application to the analysis of transcriptomics data from RNA sequencing. We emphasize the critical importance of high–quality reference profiles for enhancing the accuracy of the discussed deconvolution methods, which is essential to determine cellular compositions in complex biological samples. To ensure the robustness of our work, we have applied a rigorous selection process following the Preferred Reporting Items for Systematic Reviews and Meta–Analysis (PRISMA) guidelines. Through the review process, we have identified several key research gaps, highlighting the necessity for standardized methodologies and the improvement of the interpretability of the models. Overall, we present a comprehensive, up to date overview of the different methodologies, datasets, and findings associated with DL–driven deconvolution tools, paving the way for future research and emphasizing the value of collaboration between computational and biological sciences.

## Graphical abstract

**Figure:**
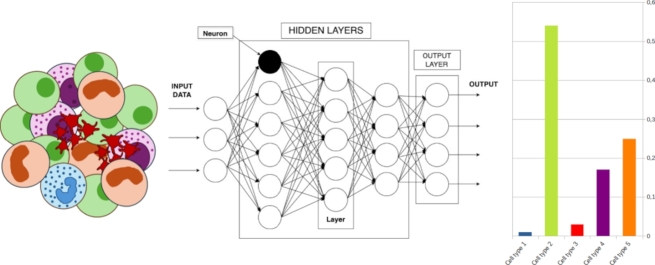

## 1 Introduction

Transcriptomics data offer a detailed view of gene expression, enabling researchers to explore cellular processes with exceptional precision. Analyzing the transcriptome is essential for identifying transcriptional isoforms, splicing events, and non-coding RNA (ncRNA) species, thereby revealing key regulatory mechanisms involved in cellular differentiation, adaptation, and pathological responses [1]. These insights are crucial not only for understanding fundamental biology but also for advancing clinical applications, including biomarker discovery, treatment evaluation, and the development of precision medicine strategies. Furthermore, epigenetic modulation and alternative splicing significantly enhance transcript diversity, shaping cell-specific functions and phenotypic outcomes.

In biology, deconvolution refers to the computational estimation of cell-type proportions within complex tissue samples using bulk gene expression data. This approach is essential for unraveling tissue composition and understanding how distinct cell populations contribute to health and disease. By resolving cellular mixtures, deconvolution provides critical insights into the tissue microenvironment, facilitating the identification of disease-associated cells, monitoring immune infiltration in cancer, and characterizing inflammatory or degenerative states. Its increasing use in biomarker discovery and targeted therapy development highlights its growing impact in both biomedical research and clinical practice.

Given the complexity of transcriptomic data, deconvolution methods have emerged as essential tools for disentangling cellular heterogeneity, particularly in bulk RNA-seq analysis. In biomedical research and therapy development, cellular deconvolution is a key tool for characterizing the cellular composition of tissues, enabling a more detailed analysis of transcriptomic profiles in bulk RNA-seq, DNA methylation, and spatial transcriptomics studies. While specific biomarkers are primarily identified through next-generation sequencing, epigenetic profiling, and proteomic analyses, deconvolution helps contextualize their expression within different cell types, aiding in disease characterization. In oncology, for instance, biomarker identification has enabled patient stratification in breast cancer [2], melanoma [3], and lung cancer [4], facilitating personalized treatments and improving clinical outcomes. Deconvolution contributes to these advances by providing insights into immune cell infiltration, tumor heterogeneity, both of which are critical factors in disease progression and therapeutic response and neurodegenerative conditions [5].

The high time and cost demands of detailed experimental approaches, such as single-cell RNA sequencing (scRNA-seq) or fluorescence-activated cell sorting (FACS), have led researchers to explore alternative strategies for characterizing cellular composition. Although scRNA-seq provides high-resolution insights, its cost—ranging from approximately USD 420 to over USD 2,250 per sample, depending on the platform and library complexity—makes it prohibitive for large-scale or routine studies. In contrast, bulk RNA-seq is substantially more affordable, with estimated costs per sample ranging from USD 37 (e.g., MERCURIUS BRB-seq, 5M reads) to USD 114 (e.g., Illumina TruSeq, ≥25M reads). These differences highlight the practical value of computational deconvolution methods, which enable researchers to infer cell-type-specific information from bulk RNA-seq data without relying on more expensive or labor-intensive techniques.

As transcriptomic datasets continue to grow in complexity and scale, Artificial Intelligence (AI) has emerged as a powerful tool for extracting meaningful insights [6]. In particular, deep learning (DL) has driven significant innovation in computational biology, offering more accurate and flexible deconvolution models [7], [8], [9].

Classical deconvolution algorithms—such as linear regression, support vector regression (e.g., CIBERSORT [10]), non-negative least squares (NNLS) (e.g., EPIC [11], quanTIseq [12]), and enrichment scoring (e.g., xCell)—typically rely on the assumption that bulk RNA-seq profiles are linear combinations of cell-type-specific signatures. While these methods are computationally efficient and interpretable, they often struggle to model complex, nonlinear relationships, are sensitive to batch effects, and depend heavily on the quality and completeness of reference signatures. These limitations hinder the detection of subtle differences between similar cell types and the identification of rare populations in heterogeneous samples.

Deep learning models, typically implemented as deep neural networks, are hierarchical architectures composed of multiple layers designed to learn progressively abstract representations from the input data [13]. These models aim to overcome the challenges faced by classical approaches by capturing non-linear dependencies, handling technical noise, and generalizing across different tissues and datasets [14]. Although their interpretability remains limited, their performance in complex deconvolution tasks often surpasses that of classical methods.

Beyond deconvolution, deep learning has also enabled major advances in other areas of biomedicine. In genomics, it has been used to predict the effects of noncoding variants directly from sequence data [15]. In clinical settings, DL models have achieved expert-level performance in medical image classification [16]. In drug discovery, AI-powered models are being used for virtual screening and compound prioritization [17]. In systems biology, graph neural networks have been applied to predict drug interactions and adverse effects through biological network modeling [18].

This systematic review analyzes the latest tools that have already revolutionized the field, similar to how AlphaFold [19], CellProfiler [20], and DeepVariant [21] have transformed their respective domains. The primary goal of this work is to provide a comprehensive review of state-of-the-art deep learning-based deconvolution methods applied to transcriptomics data. We review recent advances, key methodologies, applications, and ongoing challenges, offering a curated list of relevant publications that serve as essential reading for researchers entering the field. We also include a statistical analysis of key features across studies to highlight trends and gaps. The innovation of this work lies in presenting, to the best of our knowledge, the first up-to-date and comprehensive review of these tools in the context of transcriptomics data. We offer a curated selection of relevant papers that should be considered essential reading for anyone entering this field, alongside a statistical analysis of the most relevant features of each study.

The remainder of the paper is organized as follows: In Section 2 reviews the mathematical foundations of convolution, traditional approaches to transcriptomic data analysis, and the main deep learning models commonly applied in this context. This section also describes the PRISMA methodology followed for the systematic literature search, including the selection criteria and the key research questions guiding the review. Section 3 presents the main findings, supported by a comprehensive set of tables and figures that summarize the most relevant data. Section 4 offers a critical discussion of these results, addressing the current challenges in the field and outlining potential future directions. Finally, Section 5 summarizes the key conclusions drawn from the review and highlights the main open challenges.

## 2 Background

### 2.1 Traditional deconvolution methods

Before delving into the explanation of classical methods, it is important to emphasize that this review specifically focuses on cell type deconvolution approaches that leverage Artificial Intelligence (AI) techniques. While traditional statistical methods have laid the foundation for bulk transcriptomic deconvolution and remain widely used, they fall outside the primary scope of this article. For a comprehensive overview of classical methods—including linear regression, non-negative least squares (NNLS), matrix factorization, and marker gene-based strategies—we refer readers to the thorough review by Avila Cobos et al. [22], which systematically examines their assumptions, strengths, and limitations across various tissues and benchmarking datasets.

Briefly, traditional deconvolution methods typically rely on predefined signature matrices or selected marker genes. They are appreciated for their interpretability, computational efficiency, and effectiveness in well-characterized tissues. However, they may struggle with complex or heterogeneous samples and with adapting to dynamic transcriptional states. However, they often face challenges when dealing with complex or heterogeneous samples, as well as in adapting to dynamic transcriptional states. In contrast, this review emphasizes recent advances in AI-driven approaches, focusing on model architectures, input data requirements, and evaluation methodologies.

From a mathematical perspective, the term “deconvolution” originates from the inverse operation of convolution. In functional analysis, convolution is a well-defined process that combines two functions into a third, typically representing a filtered or mixed signal. Deconvolution aims to recover the original input signal from the convolved output, given knowledge of the filtering function.

Analogously, in transcriptomic deconvolution, bulk tissue samples are considered as mixtures of gene expression signals from various constituent cell types. Classical deconvolution models assume that the bulk expression profile can be approximated as a linear combination of cell-type-specific gene expression signatures, with the coefficients of this combination corresponding to the cellular proportions we aim to estimate. This assumption can be described by the equation

(1) $$\overset{\to }{X}=H\overset{\to }{W}+\overset{\to }{E},$$

where $\overset{\to }{X}$ is a one-dimensional vector representing the expression of a single bulk sample that needs to be deconvolved, *H* is the gene signature matrix (with dimensions g×c), $\overset{\to }{W}$ encodes the cell type proportions, and $\overset{\to }{E}$ is a noise term that captures the variability and uncertainty in the observed measurements. Typically, the elements of $\overset{\to }{X}$, *H*, and $\overset{\to }{W}$ are non-negative, and the cell type proportions must add up to one.

When deconvolving more than one bulk sample, these variables become matrices: $X\in R^{g\times n}$, $H\in R^{g\times c}$, and $W\in R^{c\times n}$, where *g* is the number of genes, *c* the number of cell types, and *n* the number of samples.

Traditional deconvolution tools are based on probabilistic models that rely on prior knowledge about the nature of the samples. These tools utilize an input matrix or vector, $X_{m\times n}$, that represents bulk RNA or DNA methylation data, where *m* is the number of genes or CpG (regions where a cytosine is followed by a guanine in the DNA sequence) sites and *n* denotes the number of samples. Cellular deconvolution methods can be classified into two main categories based on the amount of prior knowledge required: reference-based and reference-free approaches.

Reference-based algorithms require signature matrices, denoted as $H_{m\times k}$, representing gene expression or DNA methylation profiles of specific cell types. In this case, *m* is the number of marker genes, and *k* is the number of cell types. These signature matrices are typically derived from epigenomic or transcriptomic data of biologically similar samples, often obtained through flow cytometry or fluorescence-activated cell sorting (FACS). The accuracy of these methods depends heavily on the quality and relevance of the reference profiles used. Examples of tools following this strategy include CIBERSORT, ESTIMATE [23] or EPIC.

Beyond these traditional reference-based methods, several methods have emerged that can automatically infer cell type signature matrices, thus reducing the reliance on predefined references. CIBERSORTx [24], for instance, extends the original CIBERSORT framework by constructing signature matrices from scRNA-seq data through clustering and differential expression analysis. MuSiC [25] employs weighted non-negative least squares, integrating single-cell profiles while accounting for inter-subject variability. CDSeq [26] follows a fully unsupervised approach, simultaneously estimating both the signature matrix and cell type proportions from bulk RNA-seq data using a Latent Dirichlet Allocation (LDA)-based probabilistic model. These hybrid or adaptive methods serve as a bridge between reference-based and reference-free strategies, and while not based on deep learning, they illustrate the trend toward more automated, data-driven frameworks.

In contrast, reference-free methods do not require predefined signature matrices, although some may still rely on knowledge of relevant marker genes. In this setting, both the signature matrix and the cell type proportion matrix, denoted as $W_{k\times n}$, are unknown, and the objective is to estimate both matrices such that their product $H\cdot W^{T}$ closely approximates the input data matrix $X_{m\times n}$. Examples of such methods include UNDO [27] and TOAST [28].

Despite their widespread use, classical deconvolution methods have limitations, including their dependency on predefined gene signatures and strong assumptions about sample composition. For instance, the ABSOLUTE tool [29] assumes that a cancer tissue sample is composed of a given proportion of cancer cells, *α* and a proportion of healthy cells, (1−α). This assumption limits its capacity to capture broader biological variation, especially between stromal and tumoral components, which is critical for accurately estimating malignant cell proportions. Furthermore, while these methods often perform well in benchmark datasets, their generalizability and comparative accuracy on external, more diverse datasets remain open questions.

### 2.2 Deep learning models

**Fig. 1:**
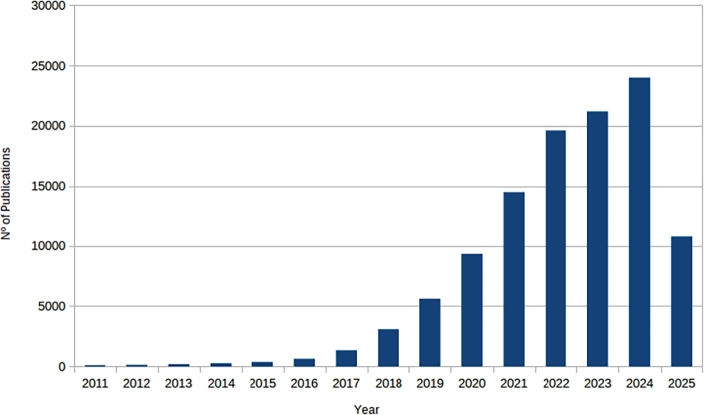
Annual publication trends of *deep learning* topic in PubMed.

In recent years, the application of deep learning in medicine and biology has experienced a significant increment, primarily due to its numerous advantages. This trend becomes evident attending to the growing number of scientific works published in specialized health science databases such as PubMed. As it is illustrated in Fig. 1, the search results for the term *deep learning* in PubMed as of May 15, 2025, indicate a notable increase in the publication volume related to this topic since 2011. The use of DL methods has a wide impact in medicine, as an increasing number of Artificial Intelligence models are recently found in both biomedical research and clinical settings [30].

Deep learning is a specialized branch of machine learning. It is characterized by its ability to extract and learn hierarchical representations of data through a succession of differentiable, and non-linear transformations. It has demonstrated its capability to automatically identify complex patterns from raw data, often outperforming classical methods in a variety of tasks, particularly in those involving unstructured and high-dimensional datasets.

Deep learning models employ stacked layers of neurons, with each layer transforming the representations it receives from the layer before. This layered approach creates increasingly processed features, though the exact nature and complexity of these features varies with the architecture and task at hand. Unlike traditional approaches that depend on manual feature engineering, deep learning models learn the relevant patterns directly from data through optimization algorithms [13]. This data-driven flexibility explains their success in various areas, including image analysis, natural language processing, and more recently, computational biology. In this latter field, we find applications such as single-cell analysis [31], cell type annotation [32], and bulk tissue deconvolution.

**Fig. 2:**
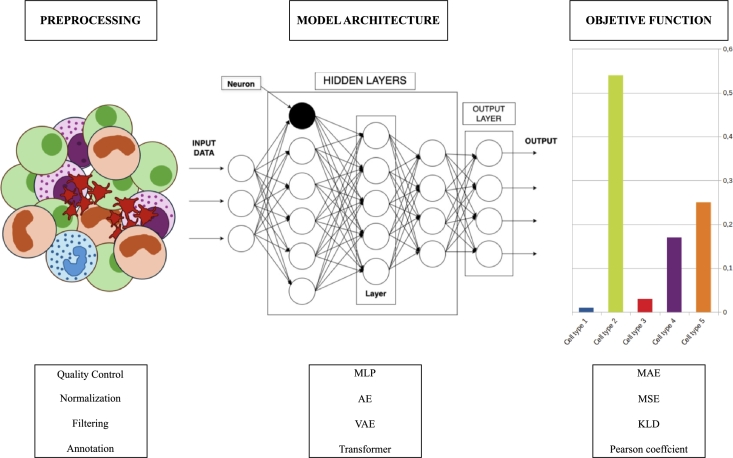
Common pipeline of an AI-based deconvolution tool.

Deconvolution tasks using AI techniques typically aim to reconstruct an original signal from noisy bulk transcriptomic data. In this context, the most used objective functions in the AI models include the commonly used *L*2 loss between the recovered signal and the ground truth, the perceptual loss within the feature space, or even Physics-informed functions when the physical processes degrading the data are known. Furthermore, the objective function typically include a proper regularization, e.g., for sparse data it is useful to add a *L*1 sparsity function to encourage the model to look only for sparse solutions in an appropriate domain. The most common pipeline of AI-based deconvolution tools is depicted in Fig. 2 as a visual guide to better understand the typical flow.

Deep learning currently encompasses a wide variety of neural network architectures specifically tailored to different tasks. The most common one, that suits a wide variety of problems at once, is the Multilayer Perceptron (MLP) architecture. Convolutional Neural Networks (CNNs) excel in image analysis, while Recurrent Neural Networks (RNNs) are suited for sequential data. Autoencoders are employed for tasks such as denoising and anomaly detection, and Generative Adversarial Networks (GANs) are used for data generation. More recently, Transformer models have gained prominence in natural language processing. Although each model was initially designed for specific types of input data, they can often be adapted or combined to address more complex problems, resulting in the development of hybrid architectures [33], [34], [35].

In more recent years, Transformer-based architectures have gained relevance in the field of Artificial Intelligence, progressively replacing traditional CNNs and RNNs thanks to their ability to capture complex and long-range relationships in sequential data through self-attention mechanisms. Originally developed for Natural Language Processing (NLP), the Transformer architecture has since been successfully adapted to other domains. For example, in computer vision, Vision Transformers (ViTs) process images by dividing them into patches and treating them as sequences, enabling effective image understanding without relying on convolutions. Similarly, in structural biology, AlphaFold [36], developed by DeepMind, uses Transformer-based architectures to accurately predict protein 3D structures from amino acid sequences.

In the field of computational biology, Transformers have shown promising results, particularly in genomics and single-cell data analysis, as they can infer complex patterns even though the data is unstructured. For instance, models such as scBERT [37] leverage Transformer-based encoders to learn representations of gene expression profiles, highlighting their potential to capture complex biological patterns. While Transformer-based deconvolution tools have not yet gained widespread adoption, their ability to handle high-dimensional, sparse data suggests they could be promising for future cell type deconvolution research.

However, only a limited subset of these models has been applied to cellular deconvolution as we will discuss in detail in the following sections.

#### 2.2.1 Multilayer perceptron (MLP)

This popular architecture was first introduced by Hinton in the 1980s [38]. MLPs are composed of multiple layers of neurons —perceptrons— arranged hierarchically. The simplest form of an MLP consists of three layers: an input layer, a hidden layer, and an output one. In contrast, deep MLPs include more than two hidden layers.

In fully connected layers, the most common architecture, every neuron receives the complete output from the previous layer. Each neuron then applies its own unique weights to this shared input, producing different weighted sums despite starting with identical data. These weighted sums pass through non-linear activation functions, enabling the layer to learn complex transformations. By having multiple neurons compute different linear combinations of the same input—combined with non-linear processing—the network can model intricate relationships between inputs and outputs. MLPs are particularly effective for modeling intricate relationships across a wide range of applications [39].

#### 2.2.2 Convolutional neural network (CNN)

Convolutional Neural Networks (CNNs) are foundational models in modern image analysis and visual recognition. Inspired by the hierarchical processing of the human visual cortex, CNNs analyze images through successive layers that detect increasingly complex features—from simple edges and textures to intricate shapes and objects. The pioneering architecture of CNNs was firstly proposed by LeCun et al. [40], marking the beginning of its practical applications.

Unlike Multilayer Perceptrons (MLPs), CNNs exploit the spatial correlations inherent in image data through convolutional operations. They apply a set of trainable filters across local regions of the input data, enabling the network to capture spatially correlated patterns. The use of pooling layers introduces the ability of finding patterns at larger distances by reducing the dimensionality of the representation while retaining its most important features.

This architecture is particularly well-suited for image data due to their inherent spatial structure, where neighboring pixels are often correlated and local patterns convey meaningful information. In contrast, CNNs are generally not suitable for gene expression data, as they lack any explicit spatial or topological structure: the ordering of genes in transcriptomic vectors is arbitrary and does not reflect any known physical proximity or functional relationships. As a result, the key assumptions that make CNNs effective for image data, namely, the presence of local dependencies and spatial continuity, are not satisfied *a priori* in gene expression matrices, which limits their utility in standard transcriptomic analysis.

#### 2.2.3 Autoencoder (AE)

An autoencoder is a deep learning architecture specifically designed to reduce the dimensionality of data through a process of encoding. It was firstly proposed by Hinton in the 1980s [41]. Although autoencoders have characteristics of both supervised and unsupervised learning, they are typically classified as unsupervised models. At their core, they consist of three key components: the encoder, which compresses the input data into a more compact, lower-dimensional representation; the bottleneck, where this compressed data is stored; and the decoder, which reconstructs the original input from the encoded representation. The different layers within each of the autoencoder components can vary in their architecture, including convolutional or MLP structures, offering then great flexibility in their design. Autoencoders are useful in a wide variety of applications, such as dimensionality reduction, data denoising, or anomaly detection, making them a powerful tool in data processing.

#### 2.2.4 Variational autoencoders (VAE)

A Variational Autoencoder (VAE) is a type of generative model designed to learn a probabilistic latent lower-dimensional representation of the input data. Unlike in traditional autoencoders, data is not encoded into deterministic latent vectors, but rather the latent space is described as a probability distribution, typically as a multivariate Gaussian distribution [42] from which new, and realistic data samples can be generated.

VAEs maximize a variational lower bound on the marginal log-likelihood of the data. Their loss function typically combines two different components: a reconstruction term that quantifies how well the model can reproduce the input data, and a Kullback–Leibler divergence (KLD) term that regularizes the learned latent distribution, encouraging it to approximate to a prior distribution. This probabilistic framework makes VAEs a specially useful model to capture biological noise and heterogeneity, which is particularly relevant in single-cell transcriptomics.

In this field, VAEs have been successfully applied to tasks such as batch correction, clustering, and data imputation, for example, in scVI [31]. More recently, their use have also been explored for deconvolution-related applications, as in DeepSEM [43] and scSemiProfiler [44], demonstrating their versatility in modeling high-dimensional, sparse, and noisy gene expression data.

Given their generative nature and probabilistic framework, VAEs form a conceptual bridge between classical autoencoders and other generative models such as GANs.

#### 2.2.5 Generative adversarial networks (GANs)

Generative adversarial networks (GANs) belong to the generative class of models. GANs consist of two neural models, the generator and the discriminator, which work together in an adversarial training process [45]. The architecture of GANs aims to learn and imitate a given data distribution. The generator is responsible for producing synthetic instances of the input data, while the discriminator evaluates these instances and decides whether they are similar enough to the input data or not. The discriminator assigns a probability of authenticity to each instance, indicating whether it is from the input distribution or synthetic. Through repeated iterations of this process, the generator learns to create synthetic data that better resembles the input distribution.

As DL models for transcriptomic deconvolution continue to diversify, a structured analysis of the existing literature is necessary. Thus, we have carried out a systematic review using the PRISMA criteria, which we detail below.

### 2.3 PRISMA strategy

A systematic literature review (SLR) is a rigorous, protocol-driven method to identify, evaluate, and synthesize research on a specific topic. Following the established guidelines [46], [47], [48], [49], our review adopts a four-step process: defining the motivation and research questions, systematically selecting relevant studies using the PRISMA criteria, critically analyzing each article, and synthesizing the findings. This synthesis highlights recent advances in deep learning-based approaches to cellular deconvolution and outlines key directions for future research. This process ensures transparency, replicability, and scientific rigor.

### 2.4 Motivation

Bulk RNA-seq captures the aggregate expression of heterogeneous cell populations, making it difficult to resolve individual cell type contributions. This review explores how deep learning models can address this challenge, offering a comprehensive overview of several current approaches. By answering the research questions in Table 1, we identify the strengths, limitations, and possible future directions in the application of AI to cellular deconvolution.

**Table 1:** Research questions guiding our systematic review.

**RQ 1: Datasets used by deconvolution models. What are their characteristics and availability?**
Finding specific transcriptomics datasets in specialized repositories can be complex. The performance of cellular deconvolution models heavily depends on their training data, requiring careful selection. Compiling and characterizing these datasets by origin, quality, and availability simplify data selection for researchers.
**RQ 2: Preprocessing techniques for transcriptomics data. Is there a standard procedure?**
scRNA-seq and RNA-seq data are often noisy and high-dimensional, negatively impacting model performance. Preprocessing these data is crucial to ensure quality and reliability for subsequent analyses. Understanding the methods used in various studies can guide researchers in handling such data effectively. Understanding the different procedures for pseudobulk formation is also essential.
**RQ 3: Targeted cell types and tissues in deconvolution models. What is the purpose of each model?**
Deconvolution models are often designed for specific purposes or clinical applications. Studying the final objectives of these tools, reveals the current research trends and highlights gaps where some cell types or conditions are underexplored providing guides for future research efforts addressing both well-studied and neglected areas.
**RQ 4: Predominant DL architectures: design, training and optimization pipelines. How are these models structured?**
Analyzing predominant AI-based deconvolution models, their architecture, along with the entire training and evaluation pipeline—including preprocessing and metrics-highlights best practices that enhance reliability, reproducibility, and model comparability.
**RQ 5: Benchmarking. How do DL models compare to other methods?**
Comparing DL techniques to SOTA methods in cellular deconvolution reveals their strengths and weaknesses. This analysis helps researchers choose the most suitable techniques for their needs. It also highlights advancements and areas for future development, driving progress in the field.
**RQ 6: Challenges and limitations of DL in cellular deconvolution. What are the emerging trends and future directions?**
Identifying the main challenges and limitations of AI approaches in cellular deconvolution, such as technical variability and model interpretability, is crucial for improving these methods. Understanding emerging trends and future directions can help guide research efforts toward overcoming these challenges.

### 2.5 Search strategy

At this stage, it is essential to define the keywords that better reflect both biological and computational perspectives. The biological terms we are considering include: (1) cellular deconvolution, (2) deconvolution, (3) bulk RNA, and (4) RNA-seq. The computational terms are the following: (5) deep learning, (6) neural networks, and (7) artificial intelligence.

**Fig. 3:**
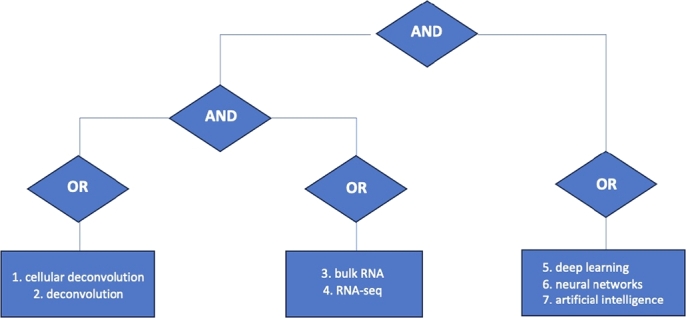
Flow diagram of the connectors employed in the PRISMA search.

Using logical operators, we structured the query as: (1 OR 2) AND (3 OR 4) AND (5 OR 6 OR 7), ensuring that the retrieved studies is pertinent to both domains. This strategy was applied to six major databases: Web of Science, PubMed, ScienceDirect, Scopus, IEEE Xplore, and SpringerLink. As MEDLINE is indexed within PubMed, it was inherently covered. The full search strategy, completed on February 3, 2025, is visualized in Fig. 3, which outlines the logical combinations of the search terms used in the systematic review.

### 2.6 Study selection and eligibility assessment

**Fig. 4:**
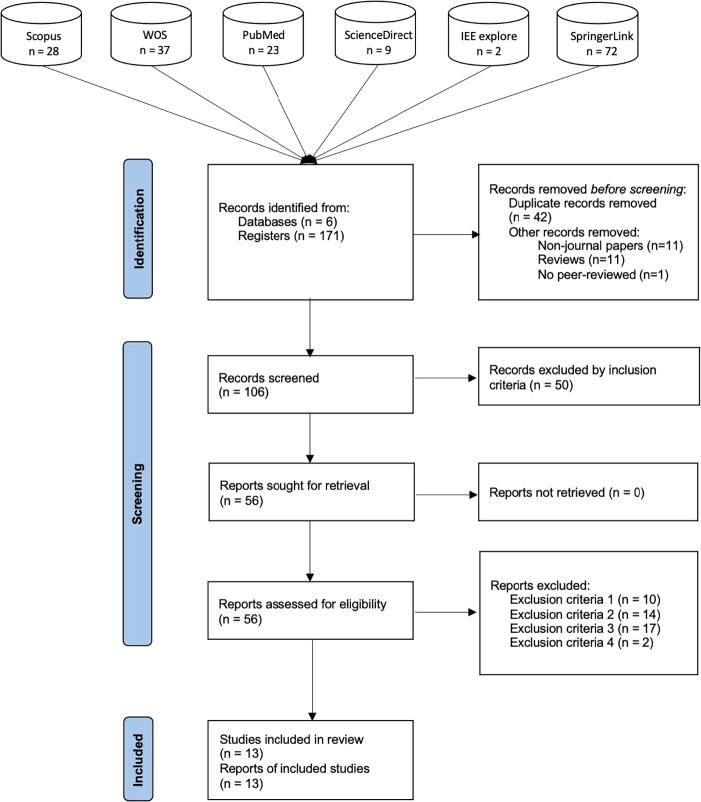
Flow diagram following the PRISMA guidelines, showing the identification, screening, eligibility assessment, and inclusion of studies in the SLR.

A set of criteria was established by a multidisciplinary team comprising computer scientists, biomedical engineers, and biotechnologists to ensure that only high-quality and relevant papers are included in our SLR. In this phase, these PRISMA guidelines were applied, as it is described in the following text and illustrated in Fig. 4.

A comprehensive search was conducted across the selected databases without publication date restrictions. Then, duplicate records were removed, and only peer-reviewed journal articles were retained. In addition, non-journal materials (e.g., books, posters, dissertations), reviews, and preprints (e.g., arXiv) were excluded so the relevance and quality of the selected works are guaranteed.

In the initial evaluation stage, only the titles and abstracts of the collected literature were assessed for relevance. This screening protocol was conducted by two independent researchers with complementary backgrounds, one in computer science and the other in biosciences, so a balanced consideration of both computational and biological perspectives is achieved. We have applied two inclusion criteria:

1. Papers that explicitly mention in their title or abstract the use of Artificial Intelligence and deconvolution tools applied to transcriptomics data.
2. Studies that use either RNA-seq or scRNA-seq data for the deconvolution task. In the case of scRNA-seq, it must be used exclusively to generate pseudobulk profiles. Studies requiring additional data types (e.g., proteomics, methylation) are not included.

Studies that did not meet these criteria were excluded, resulting in a curated selection of high-quality and relevant works for the intended scope of the review. The selected papers then underwent full-text review, and a final selection was made by applying a set of stricter exclusion criteria (see Table 2) to ensure the robustness of the review.

**Table 2:** PRISMA-based exclusion criteria for a systematic literature review.

Exclusion criterion	Reason of exclusion
Papers whose deconvolution tool requires other types of data.	Mandatory use of data types other than RNA-seq are excluded from this review. The only exception is the use of scRNA-seq data for the sole purpose of generating pseudobulk profiles. Any mandatory use of additional data modalities beyond RNA-seq and pseudobulk generation from scRNA-seq falls outside the scope of this review (bioscience criterion).
Papers that do not use DL techniques.	Some authors classify ML models within the DL category; however, ML models fall outside the scope of this review (computers sciences criteria).
Papers using DL models to process transcriptomics data but not to perform deconvolution.	Using existing deconvolution methods to subsequently design a DL-based method for other purposes is out of the scope of this review (bioscience criterion).
Papers that use existing DL-based deconvolution tools applied to a specific use case.	Out of scope (bioscience criterion).

Although all the studies contained the specified search terms, some of them only used those terms to reference other works or only mentioned them without further development. Also, studies that could not be accessed were discarded.

Only works meeting all the inclusion criteria and which pass the exclusion filters were incorporated into the final review. From all of them, comprehensive data were extracted to address the research questions.

The analysis phase provides a structured synthesis of the current landscape, focusing on: (1) extracting and organizing key information (methods, datasets, DL techniques, etc.), (2) comparing approaches to assess their strengths and limitations, (3) evaluating the quality of the study, and (4) identifying research gaps and outlining possible future directions for improving cellular deconvolution within the DL paradigm.

## 3 Results

### 3.1 PRISMA results

The search was conducted on February 03, 2025, collecting articles published up to that date. This resulted in the acquisition of 171 articles, which were subsequently downloaded to the Zotero platform for a more detailed analysis. The distribution of articles across the databases was as follows: Scopus 28, PubMed 23, WOS 37, ScienceDirect 9, IEEexplore 2, SpringerLink 72. Of the initial 171 papers, 42 were duplicates, and 23 were classified as reviews, non-journal publications, or non–peer-reviewed works. The remaining papers (n=106) were included in the screening phase. In this phase, 50 articles were eliminated because they did not meet the criterion of being a deep learning study developed to deconvolve transcriptomics data. Therefore, 56 studies were fully analyzed, of which 41 met the exclusion criteria for the review. Thus, a total of 13 papers formed the final selection. Fig. 4 shows these results in detail.

### 3.2 Summary of papers

**Fig. 5:**
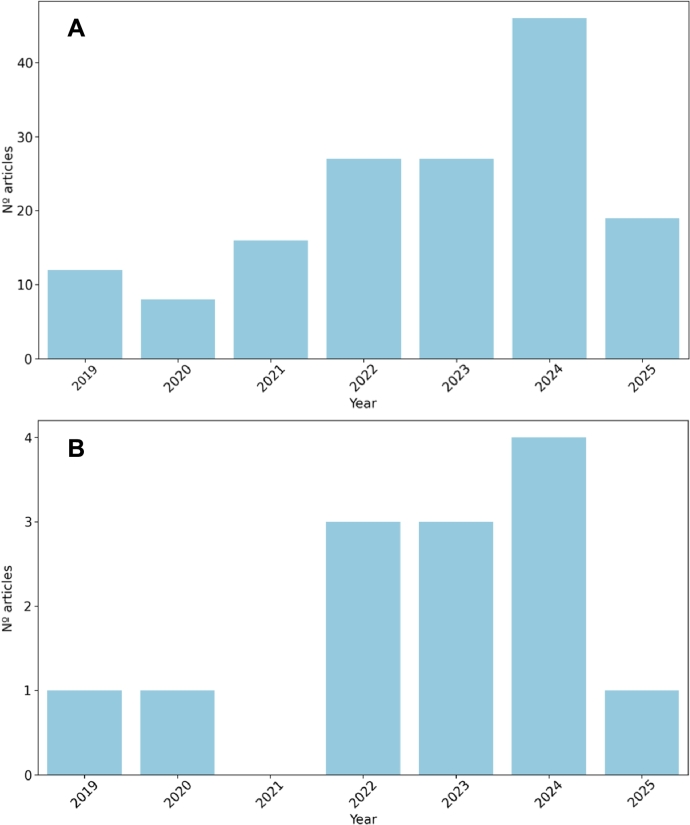
Annual number of papers on DL-based deconvolution tools per year graph.

Fig. 5 provides an updated and more comprehensive overview of the evolution of the field. Fig. 5 A illustrates the broader landscape of publications involving deep learning in transcriptomic deconvolution, including not only core tool developments but also benchmarks, reviews, and methods applying DL to related tasks. This expanded view highlights the growing interest and diversification of research in this area, with a notable increase in activity over recent years. Fig. 5 B focuses specifically on the subset of publications that were ultimately included in this review, offering a clearer view of the selected corpus and its temporal distribution.

It is also important to note that this is a relatively new and highly specialized research area, where methodological development often requires substantial biological validation and access to high-quality data. As such, the rate of publication may appear modest when compared to more mature fields. However, the diversity and technical sophistication of recent contributions indicate a clear upward trend in both interest and innovation.

The following paragraphs provide a general summary of the key highlights from the selected papers. The most relevant information has been compiled in Table 3, Table 4. Table 3 outlines the biological targets of each study and the approaches used for transcriptomics data processing. Meanwhile, Table 4 focuses on critical aspects of the DL models, including evaluation metrics, selected hyperparameters, and implementation frameworks.

1. Torroja and Sánchez-Cabo, 2019 introduced a novel method, Digitaldlsorter, for deconvoluting bulk RNA-Seq based on DL models. Digitaldlsorter is focused on the deconvolution of the immunogenic environment in breast (BC) and colorrectal (CRC) cancer samples [50].
2. Menden et al., 2020 reported Scaden, a model composed of three MLPs designed for the deconvolution of bulk transcriptomic data across various tissues, including the pancreas, brain, ovarian tissue, and PBMCs, in different species and sequencing technologies [51].
3. Zhendong et al., 2022 described a deconvolution model based on a CNN, Autoptcr, focusing on the study of the main cell types found in PBMC samples [52].
4. Lin et al., 2022 propose a pipeline called DAISM-DNN, which employs a novel in silico data augmentation strategy (DAISM) together with a MLP model to deconvolute samples from PBMC data [53].
5. Chen et al., 2022, pioneered the use of an autoencoder model for the deconvolution and generation of Gene Expression Profiles from RNA-seq samples. Their approach, TAPE, demonstrates the ability to deconvolve samples across various species and multiple organs or tissues [54].
6. Chiu et al., 2023, designed a deconvolution pipeline (HASCAD) that combines Harmony-Symphony correction with a three MLP structure of three hidden layers. This model is applied to investigate the impacts of immune cell heterogeneity on therapeutic effects, especially in cancer immunotherapy [55].
7. Charytonowicz et al., 2023, described UCDBase, an MLP-based deconvolution method with an extension called UCDSelect that uses reference data to enhance predictions. UCD study ischemic kidney injury, cancer subtypes, and tumor microenvironments by deconvolving 840 different cell types [57].
8. Yan et al., 2023, introduced an MLP-based DL model for tissue deconvolution of ctcRNA. This model is applied to study 15 different tissues and the migration of ctcRNA in a cancer patient with metastatic tumors to explore the early detection of metastasis [58].
9. Jin et al., 2024 designed NNICE, a novel method that integrates quantile regression with DL techniques to estimate cell type abundance and its variability from bulk RNA-seq data, providing an efficient and reliable solution for PBMC samples deconvolution [59].
10. Huang et al., 2024, developed DeepDecon, an iterative DL-based deconvolution tool designed to estimate the fraction of malignant cells in bulk RNA-seq samples. DeepDecon is intended for use in cancer detection, recurrence monitoring, and prognosis [60].
11. Khatri et al., 2024, reported the use of two DL networks with simultaneous consistency regularization of the target and training domains known as DISSECT. It is capable of deconvolving a variety of tissues and is easily adapted to other biomedical data types [61].
12. Sun et al. (2024) investigated changes in macrophage and T lymphocyte concentrations in kidney tissue from both healthy mice and those affected by Alport syndrome, employing a hybrid model (CONVdecov) incorporating convolutional layers and attention mechanisms. [62].
13. Morgan et al. (2025) designed a tool, GBMPurity, to estimate tumor purity in glioblastomas from human tissue, using an interpretable DL model based on a simple MLP [63].

**Table 3:** Biological Attributes for Deconvolution Techniques Summarized in This Review. The scRNA-seq preprocessing section is organized into quality control and normalization steps. In the Quality Control section, several parameters are defined: min genes indicates the minimum number of genes required for a cell to pass filtering and avoid removal due to sequencing errors; max genes specifies the threshold above which a cell is flagged as a potential doublet; min cell/gen is the minimum number of cells that must express a gene for it to be retained as relevant; and max mito denotes the maximum allowable percentage of mitochondrial genes, beyond which a cell is excluded, indicating potential cell damage or death. The Cell Type Identification section details the identification of highly variable genes (HVGs), clustering of cells into types, and cell annotation. In identifying HVGs, *n* represents the number of selected HVGs, min var is the minimum variance required for a gene to qualify as an HVG, max var is the maximum gene variance allowed, and min disp is the minimum dispersion threshold. The Cell Types section lists the number of cell types being deconvolved, which aligns with the model output. The RNA-seq processing section describes data handling, typically involving normalization and filtering of low-information genes (LIG), which are defined as genes with zero expression or with expression variance below 0.1. Additionally, the number of genes input into the model is specified. ‘NS’ stands for ‘Not Specified’, indicating missing information in the main corpus, and ‘NA’ stands for ‘Not Applicable’, indicating data or categories that do not apply to the tool or analysis.

	Objective	Tissue	sc-RNA seq preprocessing			Cell type identification			Cell types	RNA-seq preprocessing/Input genes
			Tool	Quality control	Normalization	HVGs	Clustering	Annotation		
[50]	Quantify immune infiltration in CRC and BC samples	CRC, BC	Seurat (v. NS)	NS	Library Size Scaling (TPM)	NS	Graph-based approach + t-SNE	1st Classify tumor/non-tumor (RNA-seq based CNV method a) 2nd XCell b (bulk), Single R c + manual annotation	CRC:10 BC:10	Library Size Scaling (TPM)/ BC:34145 CRC:23039
[51]	Deconvolution across different tissues and species	PBMC, Ascites, Pancreas, Brain	Scanpy (v.1.2.2)	min genes = 500 min cell/gen = 5 outliers filtering (NS)	Library size scaling	*n* = 1000 min var = 0.0125 max var = 3 min disp = 0.5	PCA + Louvain clustering (lowest resolution)	Manual annotation (Seurat 2700 PBMCs marker genes)	PBMC: 5 Ascites: 6 Pancreas: 7 Brain: 7	Remove LIG + Log Transformation d + Min-Max scaling/ ∼10000
[52]	Deconvolution of basic PBMC cellular types	PBMC	NS	NA	NA	NA	NA	NA	6	Remove LIG + Log Transformation + Min-Max scaling/ 10000
[53]	Deconvolution of different PBMC and PMN cellular types through the implementation of an augmentation method	PBMC, PMN, whole blood	Seurat (v.3.1.1)	min genes = 500 min cell/gen = 5 max mito = 10% double filtering (NS)	Log Transformation	n = 2000 min var = 0.0125 max var = 3 min disp = 0.5	PCA + SNN (n neighbors = 20)	Manual annotation (CellMarker marker genes)	6	Library Size Scaling (TPM) + Log Transformation + Min-Max scaling/ all genes
[54]	Deconvolution across different tissues and species	PBMC, Brain, Pancreas, Limb, Muscle, Marrow, Lung	NS	min genes = 500 min cell/gen = 5 outliers filtering (NS)	Library size	n = 1000 min var = 0.0125 max var = 3 min disp = 0.5	PCA + Louvain clustering (lowest resolution)	Manual annotation (Seurat 2700 PBMCs marker genes)	PBMC: 12 Brain: 7 Pancreas: 5	Library Size Scaling (TPM) + Log Transformation + LVG filtering + Min-Max scaling / ∼10000
[55]	Predict immune cell fractions in liver cancer samples	PBMC, Liver	Seurat (v. NS)	NS	Library Size Transformation (CPM) + Log Transformation + H-S normalization / –	VST^e^ n=2371	PCA+UMAP	NS	15	(Library Size Scaling(TPM) + H-S norm)/ (Library Size Scaling(TPM) + Log Transformation) + VST/ 2371
[57]	Predict cell types across multiple tissues and distinguish normal from cancerous cells	889 scRNA-seq datasets	ScanpyRAPIDS (v. NS)	min genes = 200 min cell/gen = 3 max mito = 20% counts > 2 SD^f^ above log-normal mean	Library size + Log Transformation	min var = 0.0125 max var = 4 minidisc = 0.25 z-score scaling (±10)	PCA + SNN (*n* = 20) + Leiden clustering + UMAP (min dist = 0.3)	sVAE (30 dimensions + clustering (*n* = 4200) + manual annotation	840	Library size + Log Transformation + z-score scaling (±10) + Min-Max scaling + Bias introduction/ 28867
[58]	Predict the presence of tissues in bulk samples to discover cancer progression	Digestive system, Endocrine system, Lung, Skin, Kidney, Brain, Pancreas, Breast, Uterus	NS	NA	NA	NA	NA	NA	15 (tissues)	GTEx HVGs + TCGA Validation + Min-Max scaling/ 6558
[59]	Deconvolution of 5 different PBMC cellular types	PBMC	Scanpy (v. NS)	min genes = 200 max genes = 3000 max mito = 5%	Statistical model normalization (SCTransform^g^)	SCTransform method n=3000	UMAP (10 groups)	NA	5	HVGs ImmPort immune list + Top 3000 HVG/ 3000
[60]	Estimate the fraction of malignant cells on bulk RNA-seq	AML, Neuroblastoma, HNSC	Scanpy (v.1.7.2)	min genes = 500 max genes = 3000 min cell/gen = 5 outliers filtering (depends on dataset)	Library size	NS	UMAP	NS	2	LIG + TF-IDF^h^ / Library size(TMP) + Min-Max scaling/ 3000
[61]	Deconvolution of different tissues and species	PBMC, Pancreas, Kidney, Brain	NS	min genes = 200 min cell/gen = 3 max mito = 4%	NS	NS	NS	NS	Pancreas: 6 PBMC: 18 Kidney: 9 Brain: 10	Library size (CPM) + Log Transformation + Min-Max scaling/ NS
[62]	Deconvolution of mouse kidney tissue for the study of Alport syndrome	Kidney	Scanpy (v. NS)	min genes = 500 max genes = 1500 max mito = 5% total counts < 6000	NS	NS	NS	NS	4	NS / 1877
[63]	Cellular deconvolution of brain tissue to investigate Glioblastoma purity	Brain	Scanpy (v. 1.9.8) Seurat (v. 5.1.0)	GBmap: min genes = 500 total counts > 1000 max mito = 30% Neftel et al.: min genes = 200 total counts > 800 max mito = 5% doublet detection (DoubletFinder)	Library size + Log Transformation	n = 2000	Integration + PCA (n = 50)	CONICSmat^i^ (v. 0.0.0.1)	NA	$\left(\log_{2}(TPM/100)+1\right)$ / 5829

a [56].

b https://aran-lab.com/xcell2-vignette.

c https://www.bioconductor.org/packages/release/bioc/html/SingleR.html.

d $\log_{2}(X+1)$.

e Variance-stabilizing transformation.

f Standard deviation.

g https://satijalab.org/seurat/articles/sctransform_vignette.html.

h Term Frequency-Inverse Document Frequency (TF-IDF).

i https://github.com/Neurosurgery-Brain-Tumor-Center-DiazLab/CONICS.

**Table 4:** Summary of deep learning architectures, hyperparameters, training setups, and performance metrics used in deconvolution models. Each row presents details of a specific study, including its architecture, key hyperparameters (e.g., activation functions, optimizers, dropout rates), and training configurations (e.g., loss functions, training-validation split, batch size). Grid Search section shows whether a grid search has been performed to optimize the model architecture. Loss and Metrics section specifies the chosen functions for training and evaluating the models respectively. Best Result column contains the best achieved results of each tool in its benchmarking. The Implementation column specifies the programming language and deep learning framework used for each model.

	Architecture		Hiperparameters						Training		Metrics	Best Result	Implementation	
	DL model	Layers	Grid Search	Activation Function	Optimizer	Dropout	Epochs	Loss	Train / Val	Batch Size			Language	Framework
[50]	MLP	2HL	Yes	ReLU (HL) SoftMax (OL)	Adam (LR:NS)	0.25	50	KLD MAE MPE	70/30	100	Corr matrix (Digitaldlsorter/benchmark tools)	BC data highest correlation (*R* = 0.81): “STROMA” type with EPIC CRC data highest correlation (*R* = 0.86): T cells with ESTIMATE	Python (NS)	TensorFlow (NS) Keras (NS)
[51]	3 MLP	4HL	Yes	ReLU (HL) SoftMax (OL)	Adam (LR:10^−4^)	M512 (0, 0.3, 0.2, 0.1) M1024 (0, 0.6, 0.3, 0.1)	5000 (steps)	L1	Depends on the tissue	128	RMSE CCC slope *R*^2^ r	Deconvolution ROSMAP dataset: RMSE=0.06 CCC= 0.92	Python (v. 3.6.8)	TensorFlow (v. 1.10.0)
[52]	CNN	CNN (4 Conv1D + 4 MaxPooling) + MLP (1HL)	Yes	ReLU (HL)	Adam (LR:10^−3^) SoftMax (OL)	NS	2000 (steps)	MSE	4-fold cross validation	128	RMSE LCC r	Deconvolution PBMC2 dataset: RMSE=0,093 LCC=0.293 r = 0.476	NS	NS
[53]	MLP	3HL	Yes	NS	Adam (LR:10^−4^)	NS	Patience val error (10 epochs)^a^	MSE	80/20	64	RMSE CCC r	Deconvolution SDY67 dataset: RMSE < 0.04 CCC = 0.8 *r* > 0.8	Python (v. 3.7.7)	PyTorch (v. 1.5.1)
[54]	AE	Encoder: 4HL Decoder: 4HL	No	Encoder: CELU Decoder: ReLU	Adam (LR:10^−4^)	Encoder: 0.5	Training stage: 5000 (encoder) Adaptive stage: 300 steps (AE)	MAE CCC	NS	128	CCC	Deconvolution ROSMAP dataset: CCC = 0.14	Python (NS)	PyTorch (NS)
[55]	3 MLP	3HL	NS	NS(HL) SoftMax (OL)	Adam (LR:10^−4^)	NS	20	MSE r	50/50	64	MSE r	Deconvolution 9 PBMC datasets (GSM2871599-GSM2871607): MSE < 0.02 *r* > 0.6	Python (NS)	TensorFlow (v. 2.12.0)
[57]	MLP	4HL	Yes	ELU (HL) SoftMax (OL)	Adam (LR:10^−4^)	No	epochs: 50 Patience train error (4 epochs)	Sparse MSE r	80/20	256	MSE CCC r	Deconvolution on 9 real datasets: *r* = 0.68	Python (NS)	TensorFlow (v. 2.12.0) Keras (v. 2.12.0)
[58]	MLP	1HL	NS	ReLU (HL) SoftMax (OL)	Adam (LR:NS)	NS	epochs: 200 Patience val error (5 epochs)	MSE	90/10	64	MSE r	Deconvolution HTR-TASR (6 tissues): MSE < 0.02 *r* = 0.95	Python (v. 3.8.3)	Keras (v. 2.12.0)
[59]	30 MLP (6 MLP x 5 quantiles)	NS	Yes	Linear activation function (HL/OL)	NS	L2 (*λ* = 10^−4^)	NS	TiltedLoss = max⁡((qξ), ((*q* − 1)*ξ*))	90/10 (10-fold cross validation)	NS	RMSE r	Deconvolution SDY67 dataset: *r* = 0.7	Python (NS)	Keras (v. 1.0.8)
[60]	55 MLP	4 HL	Yes	ReLU (HL) SoftMax (OL)	Adam (LR:10^−4^)	0.1	Epochs: 50 Patience train loss (5 epochs)	RMSE	leave-one-out cross-validation	128	RMSE CCC R	Deconvolution real AML data: Primary (RMSE = 0.13) Recurrent (RMSE = 0.19) Beat AML (RMSE = 0.17)	Python (NS)	Keras (v. 1.0.8)
[61]	MLP	5 HL	Yes	ReLU (HL) SoftMax (OL)	Adam (LR:10^−5^)	NS	5000 (steps)	*L*_total_ = *L*_KLD_ + *λ*⁎*L*_cons_	Depends on the tissue	64	RMSE JSD r	An average improvement of 0.063 in JSD and 0.021 in RMSE	Python (NS)	TensorFlow (v. 2.7.0) Keras (v. 2.7.0)
[62]	MLP	2HL	Yes	ReLU (HL)	Adam (3 × 10^−4^)	0.4	Until convergence^b^	L1	11 cross-validation	64	RMSE MAE CCC r	Deconvolution EORTC dataset: RMSE = 0.160 MAE = 0.128 CCC = 0.743 r = 0.757 Deconvolution TCGA dataset: RMSE = 0.123 MAE = 0.102 CCC = 0.701 *r* = 0.803	Python (v. 3.10.13) R (v. 4.3.1)	PyTorch (v. 2.2.0)
[63]	Feature map construction + SE-block	1 Conv3x3 + Average pooling + MLP (2 HL) + attention mechanism	NS	Tanh (1HL) Sigmoid (2HL) SoftMax (OL)	NS	NS	NS	NS	89:11	NS	RMSE r MAE	Deconvolution of real bulk data: r = 0.86 RMSE = 0.07 MAE = 0.06	NS	NS

a Early stopping criterion.

b The average training loss failed to decrease over a sliding window of 25 batches.

#### 3.2.1 Datasets used by deconvolution models

**Fig. 6:**
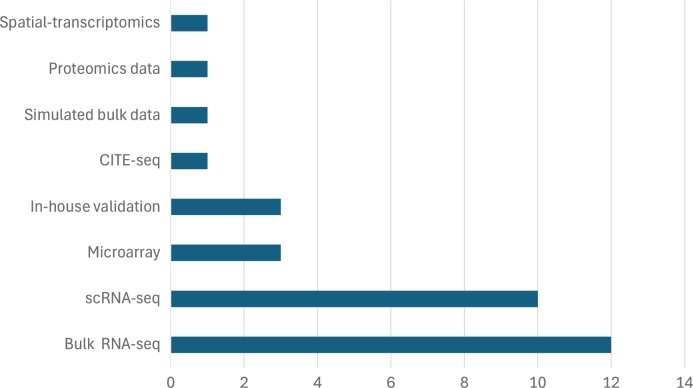
Distribution of Genomic Data Types Used in Deconvolution Studies. Simulated bulk data category refers to studies where the data are not simulated, but instead, pre-existing simulated data are used, and In-house validation category refers to studies where the data are generated or collected internally by a team or laboratory to validate and assess the model performance.

This section examines the datasets utilized in the reviewed studies, focusing on their specific characteristics. The goal is to clarify the types of data employed in developing deconvolution tools with DL, their biological origins, and the sources from which they were acquired. The first part of the analysis identifies the various data types used in the development of the deconvolution tools covered in this review. Fig. 6 illustrates the diversity of genomic data and their distribution, highlighting the number of studies in which each type is featured. Each paper, regardless of whether it employs multiple datasets of the same type or just one, is counted equally. For this section, UCDBase tool has been excluded due to its extensive dataset collection (over 1000 datasets), which makes detailed analysis unfeasible.

As we show, all studies (n=12) use bulk RNA-seq data, in consonance with the search criteria described in the methodology section. In most of the cases (n=10), scRNA-seq data is also required to generate pseudobulk data. Data from microarray technologies appears in 23% of the studies, while other types of data (spatial transcriptomics and proteomics) are used less frequently. It is also notable that three of the studies use data generated by the authors themselves.

**Fig. 7:**
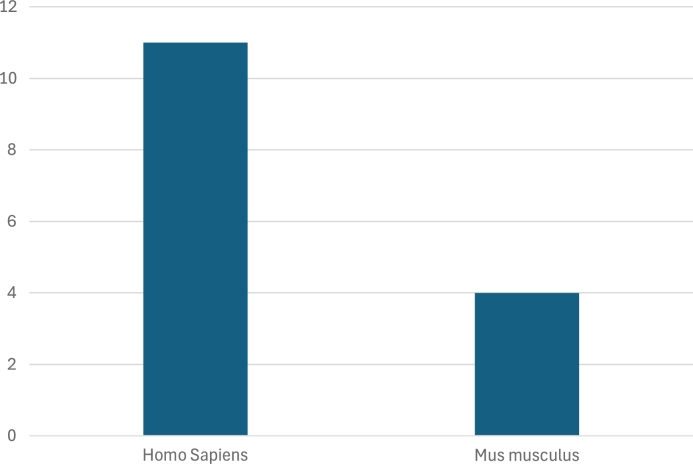
Species Distribution in Datasets Used for Deconvolution.

**Fig. 8:**
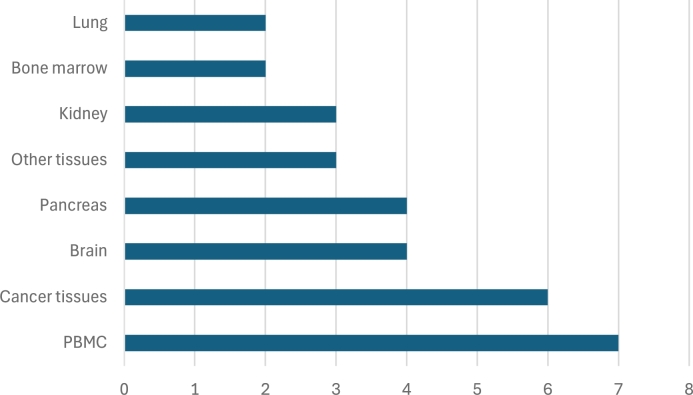
Distribution of Tissue Origins in Deconvolution Datasets.

Focusing on the predominant data types (bulk RNA-seq and scRNA-seq), the next questions concern the origin of these datasets, specifically the species and tissue types targeted for deconvolution. In Fig. 7 we show the predominance of *Homo sapiens* data compared to *Mus musculus* data, with the former one used in all studies and the latter appearing in only four out of twelve. Meanwhile, in Fig. 8 we illustrate the distribution of tissue origins for the datasets, emphasizing the predominance of data from PBMC samples, followed by cancer tissue studies, as well as pancreatic and brain tissue.

The databases and repositories used by authors to acquire data are also significant. The primary sources are NCBI-GEO,^1^ 10x Genomics,^2^ ImmPort,^3^ and TCGA.^4^ NCBI-GEO and 10x Genomics are public repositories for storing high-throughput gene expression data, with the latter specifically focused on scRNA-seq data. ImmPort is an immunology-focused repository, offering access to clinical and experimental data, while TCGA provides genomic, epigenomic, transcriptomics, and clinical data from patients across more than 20 types of cancer.

**Fig. 9:**
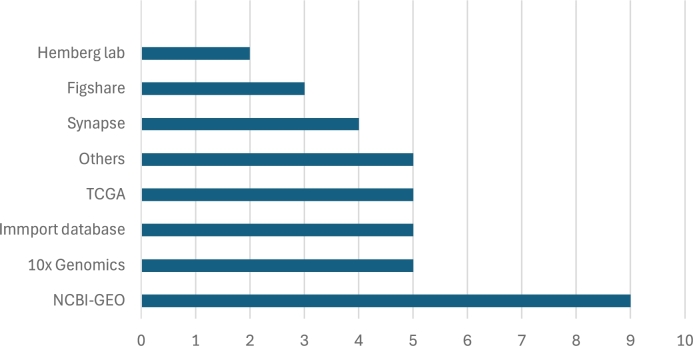
Data Repositories Used in Deconvolution Studies.

In Fig. 9, the ‘Others’ category includes databases such as Zenodo,^5^ TISCH,^6^ Single Cell Portal,^7^ Allen Brain Atlas,^8^ cBioPortal,^9^ GDC Data Portal,^10^ LinkedOmics,^11^ GTEx,^12^ CGGA,^13^ EGA^14^ and cellxgene.^15^ These are grouped together under ‘Others’ as each is mentioned in only one study.

After a detailed analysis of each article, it was found that certain datasets are more commonly used across multiple studies. In Table 5 we summarize the most frequently used scRNA-seq datasets and their characteristics, while Table 6 provides the details on each of the bulk RNA-seq datasets.

**Table 5:** Frequently Used scRNA-seq Datasets and Their Characteristics.

	Name	Citation	Organism	Platform	Tissue	Cells/individuals	Resume
[51], [53], [61]	6k PBMCs from a Healthy Donor	10x Genomics	*H. sapiens*	Illumina NextSeq 500	PBMC	5419/1	scRNA-seq data from PBMCs obtained from a healthy donor
[51], [53], [54], [61]	8k PBMCs from a Healthy Donor	10x Genomics	*H. sapiens*	Illumina Hiseq4000	PBMC	8381/1	scRNA-seq data from PBMCs obtained from a healthy donor
[51], [54], [61]	GSE84133	Baron et al., 2016 (Hemberg lab/GEO)	*H. sapiens*	InDrop	Pancreas	8569/4	scRNA sequencing of pancreatic islets from human donors (15 cellular types)
[51], [61]	Frozen PBMCs (Donor A)	10x Genomics	*H. sapiens*	Illumina NextSeq 500	PBMC	2900/1	scRNA-seq data from Frozen PBMCs obtained from a donor
[51], [61]	Frozen PBMCs (Donor C)	10x Genomics	*H. sapiens*	Illumina Hiseq2500	PBMC	9519/1	scRNA-seq data from Frozen PBMCs obtained from a donor
[51], [61]	Segerstolpe	Segerstolpe et al, 2016 (Hemberg lab)	*H. sapiens*	Smart-Seq2	Pancreas	25525/10	scRNA-seq samples of endocrine and exocrine cell types of the human pancreas in healthy and T2D (Type 2 diabetes) individuals
[51], [54]	GSE87544	Chen et al., 2017 (Hemberg lab/GEO)	*M. musculus*	Drop-seq	Mouse Brain	14437/1	scRNA-seq on hypothalamus of mice under 2 conditions (normal condition or food deprivation)

**Table 6:** Frequently Used Bulk RNA-seq Datasets in Deconvolution Studies.

	Name	Citation	Organism	Platform	Tissue	Individuals	Resume
[51], [53], [54], [59], [61]	SDY67	Immport data-base Zimmermann et al., 2016	*H. sapiens*	–	PBMC	159	RNA-seq data for B-cell immune responses study in rheumatoid arthritis and healthy controls.
[51], [53], [54], [59], [61]	GSE107011	Xu et al., 2019 GEO	*H. sapiens*	Illumina HiSeq 2000 (Homo sapiens)	PBMC whole blood	4 (whole blood) 13 (PBMC)	RNA-seq data of 29 cell types from PBMC and blood samples of Chinese Singaporean individuals.
[51], [54], [61]	ROSMAP	Bennett et al., 2019 Synapse	*H. sapiens*	–	Brain	508	RNA-seq data from brain tissue of deceased participants for the study of neurodegenerative disorders. Patients are classified as having no cognitive impairment, mild cognitive impairment or dementia, and five other conditions, such as stroke and parkinsonism.
[51], [52], [59]	Kevin	Figshare	*H. sapiens*	–	PBMC	–	In silico dataset from 6K,8K, Donor A, Donor C.

#### 3.2.2 Preprocessing techniques for transcriptomics data

Before deconvoluting transcriptomics data, it is important to consider the preprocessing methods used, as this is a crucial step. In this section, we explore the methodology followed in the preprocessing of scRNA-seq, bulk RNA-seq, and pseudobulk data generation in each of the selected studies we reviewed.

Depending on the specific stage of the workflow within each study, such as constructing pseudobulk data or annotating cell types, the preprocessing of scRNA-seq data can generally be divided into two main stages. The first stage focuses on quality control and normalization. Quality control involves identifying and removing defective data, such as low-quality cells, doublets, and genes with low expression, which could otherwise lead to biased results. Normalization adjusts for systematic and technical variations, such as differences in sequencing depth across cells, enabling accurate comparisons of cells and genes. Effective normalization methods are essential for ensuring that differences observed in gene expression reflect biological variability rather than technical artifacts. After completing these steps, the data are ready for the pseudobulk formation. In Table 3 we outline the procedures employed in each of the studies we reviewed. It is interesting to note that although some studies, such as Scaden, TAPE and DISSECT, work with the same datasets, they present a lack of consistency in the criteria used for quality control. For example, the selected thresholds can significantly differ in terms of the minimum number of genes per cell, the minimum number of cells required for a gene to be considered significant, or the maximum allowable proportion of mitochondrial genes. While the precise effects of these differing criteria remain still unclear, they do influence in the formation of pseudobulk samples and, consequently, in the training of the models.

The second stage is particularly important for cell annotation, which is part of six out of the ten studies that use scRNA-seq data. This processing continues with the calculation of highly variable genes (HVGs), which are then compared to marker genes from the studied cell types. These markers can come from databases like CellMarker^16^ for manual annotation or be used for semiautomated annotations through tools like scClassify^17^ or CHETAH,^18^ which usually require some manual checking afterwards [64]. The criteria for selecting HVGs can vary widely, encompassing thresholds for variance and the total number of genes considered. A larger pool of HVGs generally provides more information, leading to more accurate annotations. Furthermore, differences in clustering criteria are evident, particularly with regard to resolution; higher resolution often leads to a greater number of clusters, resulting in the identification of more cell types and a higher level of detail.

Once the scRNA-seq data has been processed, the next crucial step is creating pseudobulk samples. This process is vital because DL models require substantial amounts of bulk RNA-seq data to be properly trained. While many real RNA-seq datasets are accessible to the scientific community, they often suffer from limited size or unreliable annotations. In Table 7 we highlight the key considerations in this process, such as the total number of cells (*N*) in each pseudobulk sample, the included cell types (*C*), or the method used to calculate cell fractions. The “Bias” column refers to the intentional introduction of variability into the training data through strategies such as gene masking or the addition of Gaussian noise. These techniques are not employed as conventional data augmentation methods, but rather as mechanisms to simulate diverse training conditions and to assess the impact of input perturbations on the model robustness and generalization power. The “Intersample Formation” column indicates whether pseudobulk samples are generated from cells originating from a single individual (intrasample) or if they are aggregated across multiple individuals (intersample).

**Table 7:** Criteria for Pseudobulk Sample Formation in Reviewed Articles. “Bias” column indicates whether artificial variability was introduced into the training data (e.g., via gene masking or noise injection) to simulate diverse conditions. “Intersample Formation” column specifies whether pseudobulk samples were constructed from single-individual (intrasample) or multi-individual (intersample) cell pools.

	*N* (number of cells)	*C* (cell types)	F_c (cell fractions)_	Bias	Intersample Formation	Pseudobulk Samples
[50]	100	CRC: 10 BC: 10	Truncated uniform distribution and Dirichlet distribution	No	Yes	17550 (all datasets)
[51]	500	PBMC: 5 Ascites: 6 Pancreas: 7 Brain: 7	Random generation with uniform distribution	Yes	No	32,000 PBMC 14,000 pancreas 6000 human brain 30,000 mouse brain
[53]	500	6	Dirichlet distribution	No	NA	16,000
[54]	NS	Limbs: 6 Bone marrow: 7 Lung: 9 PBMC:12 Brain: 7 Pancreas:5	Dirichlet distribution with prior knowledge	Yes	No	5000
[55]	NS	15	Random generation with uniform distribution	NS	No	16000
[57]	1–10,000	1–32	Random generation	Yes	Yes	10 M
[59]	500	5	Dirichlet distribution	No	No	11000
[60]	3000	2	Uniform distribution	Yes	No	3000
[61]	100	Pancreas: 6 PBMC: 18 Kidney: 9 Brain: 10	Uniform distribution	No	No	1,000*XC*
[62]	200–400	NA	Random generation	No	No	197
[63]	15	4	Random generation	No	No	4400

It is worth noting that for Digitaldlsorter, not only multiple pseudobulks are created, but also individual cellular profiles are simulated using the ZinbWabe tool,^19^ resulting in in silico samples composed of both real and simulated cells. Additionally, within the GBMPurity tool, pseudobulks are dynamically generated during the training process, until it reaches convergence. Once the model has converged, no additional pseudobulk samples are generated.

Lastly, both real and synthetic RNA-seq data require preprocessing before being analyzed by deconvolution tools. This step includes normalizing the count table and selecting the most informative genes, essentially HVGs that will serve as the inputs for the model. As in the case of scRNA-seq data preprocessing, in Table 3 we show the variation in normalization and gene selection criteria for the bulk scenario. This is particularly important as it defines how much information the model will have access for deconvolution. There is a considerable disparity between models: for instance, Scaden uses approximately 10,000 genes, while CONVdeconv works with fewer than 2,000 genes.

Although we do not provide a systematic evaluation of the impact of preprocessing steps on deconvolution performance, their potential influence is important to acknowledge. For example, different normalization strategies applied to scRNA-seq data can alter the scale and distribution of gene expression values, thereby affecting the model's input and learning dynamics. Likewise, the number and identity of genes selected for training define the amount and specificity of information available to the model. While including more genes may improve resolution, it can also introduce noise if not carefully filtered.

A common strategy is to normalize RNA-seq gene expression data to a range between 0 and 1 using min–max scaling, as this has been shown to improve the performance of DL models.

Additionally, the construction of pseudobulk samples, whether derived from individual donors or pooled across different individuals, can shape the biological variability represented in the training data. Aggregating across samples may obscure individual-specific expression patterns, potentially limiting the model's ability to generalize.

These considerations highlight the critical role of harmonized and transparent preprocessing pipelines, especially when comparing or applying deconvolution models across datasets. We believe that future efforts to systematically quantify the impact of individual preprocessing decisions on model performance would provide valuable insights and help advance best practices in the field.

It is also important to highlight the software tools used during preprocessing. Out of the 13 papers we have reviewed, six employed Scanpy^20^ (Python), four used Seurat^21^ (R), and the remaining studies did not specify the utilized tools.

#### 3.2.3 Targeted cell types and tissues in deconvolution models

Understanding the specific objectives and use cases for which each deconvolution model is developed is essential for selecting the most appropriate tool for a specific study. These models are typically divided into two main categories: those aimed at addressing specific clinical challenges and those developed to overcome computational constraints. Users choose the tool that best fits their biological or analytical goals, taking into account both their specific objectives and the origin of the data. This distinction provides valuable guidance in making an informed decision.

The first category includes general purpose models such as DeepDecon, aimed at detecting tumor cells; Digitaldlsorter, which performs deconvolution across various cancer types; and Yan et al., 2023 designed a tool for monitoring cancer progression. Additionally, there are more specialized models: [62] focuses on immune system-related changes in mouse Alport syndrome, while GBMPurity was developed to estimate tumor purity in glioblastomas.

#### 3.2.4 Clinical purposes

**Fig. 10:**
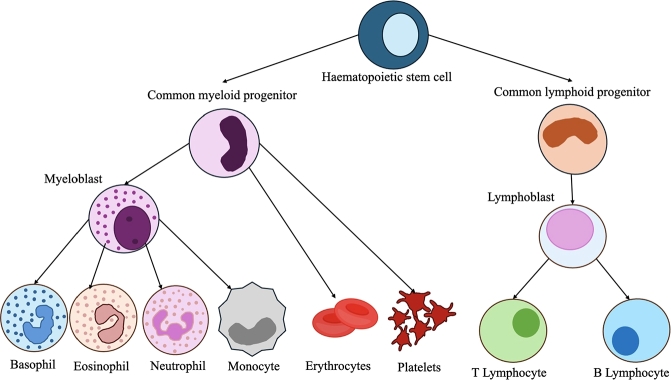
Overview of the different cell types that arise from hematopoietic stem cells through the process of hematopoiesis. The diagram illustrates the differentiation pathways leading to the formation of key blood cell lineages, including myeloid and lymphoid progenitors. The myeloid lineage gives rise to erythrocytes, platelets, monocytes, and granulocytes (basophils, eosinophils, and neutrophils), while the lymphoid lineage differentiates into T and B lymphocytes.

In Table 3, we show the different types of tissues that are used for training and evaluation, as well as the number of cell types that each model can identify in each context. While deconvolution models can be applied to a wide range of tissues, many are commonly trained or benchmarked on blood-derived samples, particularly Peripheral Blood Mononuclear Cells (PBMCs). This frequent use of PBMCs is not incidental, as they are of broad interest in biomedical research due to their central role in the immune system [65]. The relative proportions of different PBMC subtypes can provide valuable information about immune status, disease progression, or response to therapies—making them especially useful in contexts such as cancer, chronic infections, or immunological disorders. To illustrate the complexity of PBMC cellular composition and the challenge it poses for deconvolution, we present in Fig. 10 a comprehensive overview of PBMC origin and subtypes.

In addition to immune system cell types, some models, such as Digitaldlsorter and DeepDecon, focus exclusively on the study of cancerous samples. The first focuses on the deconvolution of tissues affected by two different types of cancer, offering detailed insights into the tumor state through cellular proportions, while the second aims to estimate the proportions of cancerous cells more generally. These models analyze tumor heterogeneity and its progression, including metastasis.

**Fig. 11:**
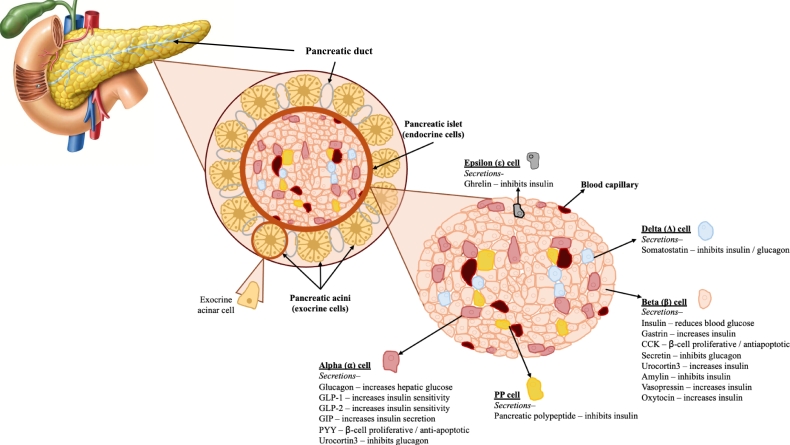
Cellular Heterogeneity in Pancreatic Tissue. The diagram illustrates the structural organization of the pancreas, highlighting key components such as the pancreatic duct, pancreatic islets, and acini. It also depicts the diverse cell types within these structures, emphasizing the complexity of pancreatic tissue architecture.

Other tissues that are examined by several of these models include pancreatic tissue, whose graphical depiction can be seen in Fig. 11. Analyzing cellular heterogeneity is of vital importance to study diseases such as diabetes or pancreatic cancer. Furthermore brain tissue has received special attention due to its relevance in research for neurodegenerative diseases such as Alzheimer and other kind of dementias.

In the context of pancreas analysis, current deconvolution efforts primarily focus on endocrine cell types, such as alpha, beta, delta, and epsilon cells. While these efforts are valuable for understanding normal pancreatic function, they often overlook the broader cellular landscape of pancreatic tissue, especially relevant in cancerous contexts. A more comprehensive approach should also include other key cell types, such as stromal, immune, and malignant cells, to fully capture the complexity of the tumor micro-environment. These additional cell types play a critical role in disease progression and influence the interactions with endocrine cells. The lack of tools capable of deconvolving the full spectrum of cellular populations limits our ability to fully understand the cellular heterogeneity in pancreatic ductal adenocarcinoma (PDAC), for example.

On the other hand, in the context of clinical applications, the granularity of deconvolution models is crucial. Significant differences emerge depending on the tool used, particularly in their resolution when applied to PBMC subclasses. Some tools, such as Autoptcr, can only distinguish two types of T lymphocytes and a single class of B lymphocytes within lymphoblast-derived cells. Other tools, like HASCAD and NNICE, are limited to identifying five PBMC cell types. In contrast, models such as TAPE can differentiate up to 12 distinct PBMC populations, while DISSECT stands out by resolving up to 18 cell types. Higher-resolution methods provide a more detailed characterization of cellular subpopulations, facilitating the identification of maturation stages, functional states, and a more nuanced understanding of immune heterogeneity. This enhanced granularity can be critical for clinical decision-making, particularly in monitoring disease progression and therapeutic responses.

#### 3.2.5 Biological computational challenges

The second category includes tools developed to address computational challenges without being tied to a specific biological or clinical context. These solutions encompass novel data generation strategies, such as the pipeline proposed by DAISM-DNN, and the application of innovative DL architectures like TAPE, previously unexplored in this domain. These tools aim to offer adaptable solutions applicable to a wide variety of tissues and cell types, as demonstrated in UCDBase.

The DAISM (Data Augmentation through In Silico Mixing) method tries to enhance the training of deep learning models for estimating cell type proportions from bulk RNA-seq data. To overcome the challenge of limited training data, DAISM generates synthetic samples by in silico mixing of calibration datasets with known cell type fractions. Starting with a small set of real samples, the method creates randomized mixtures of cell types, enabling the development of prediction models that are tailored to specific datasets. This approach helps to avoid cross-platform variability by training exclusively on the augmented dataset.

In HASCAD, the authors propose a versatile model capable of deconvoluting cell types across various transcriptomics data modalities, facilitating the analysis of complex cellular mixtures from different tissues. This approach is particularly valuable for understanding cellular composition and dynamics in diverse biological scenarios. By handling heterogeneous samples, the model offers significant advantages for research into conditions such as idiopathic pulmonary fibrosis, type II diabetes mellitus, and multiple sclerosis.

Table 8 details the cell types by tissue that the tools reviewed here are capable of deconvolving.

**Table 8:** Comparison of deconvoluted cell types across different tissues and tools. The cell types in parentheses are subtypes of the higher-level cell type identified by the tool.

	CRC	BC	PBMC	Pancreas	Ascites	Brain	Kidney	Others
[50]	Colon epithelial tumor cells, Healthy epithelial cells, Fibroblasts, CD4+ T cells, CD8+ T cells (Gzmb+/-), Monocytes, Macrophages, Germinal center B cells, Peripheral B cells	CD4+ T memory cells, CD4+ T regulatory cells, CD4+ T helper cells, CD8+ T cells, Monocytes, Germinal center B cells, Plasmacytoid dendritic cells, Memory B cells, Stroma (Healthy fibroblasts, Endothelial cells, Epithelial cells), Tumor (HER2, Luminal A/B, TNBC)	Monocytes, CD8+ T cells, CD4+ T cells, Natural Killer cells, B cells	–	–	–	–	–
[51]	–	–	Monocytes, CD8+ T cells, CD4+ T cells, Natural Killer cells, B cells	Alpha cells, Beta cells, Gamma cells, Delta cells, Acinar cells, Ductal cells, Endothelial cells	Monocytes, CD8+ T cells, CD4+ T cells, Dendritic cells, Fibroblasts, Carcinoma cells	Oligodendrocytes, Excitatory neurons, Inhibitory neurons, Astrocytes, Endothelial cells, Oligodendrocyte precursors, Microglia	–	–
[52]	–	–	Monocytes, CD8+ T cells, CD4+ T cells, B cells, Natural Killer cells	–	–	–	–	–
[53]	–	–	Monocytes, CD8+ T cells, CD4+ T cells, Natural Killer cells, B cells, Neutrophils	–	–	–	–	–
[54]	–	–	Natural Killer cells, Monocytes, Myeloid dendritic cells, Plasmacytoid dendritic cells, Naïve B cells, Memory B cells, MAIT cells, Naïve CD8+ T cells, Naïve CD4+ T cells, Non-Naïve CD4+ T cells, Non-Naïve CD8+ T cells, CD4+ T regulatory cells	Alpha cells, Beta cells, Gamma cells, Delta cells, Epsilon cells	–	Oligodendrocytes, Excitatory neurons, Inhibitory neurons, Astrocytes, Endothelial cells, Oligodendrocyte precursors, Microglia	–	–
[55]	–	–	Natural Killer cells, Naïve B cells, Memory B cells, Plasmacytoid dendritic cells, Activated dendritic cells, Monocytes (Monocytes14, Monocytes16), Naïve CD8+ T cells, CD8+ Effector T cells, CD8+ Memory T cells, Naïve CD4+ T cells, CD4+ Memory T cells, CD4+ T regulatory cells, Hematopoietic stem cells, Megakaryocytes	–	–	–	–	–
[59]	–	–	CD8+ T cells, CD4+ T cells, B cells, Natural Killer cells, Myeloid cells	–	–	–	–	–
[60]	–	–	–	–	–	–	–	Malignant / Non-malignant
[61]	–	–	B cells (Exhausted B cells, Non-Switched Memory B cells, Naïve B cells, Switched Memory B cells), Monocytes (Monocytes C, Monocytes I, Monocytes NC), Natural Killer cells, Neutrophils, Plasmablasts, CD4+ T cells (Naïve CD4+ T cells, CD4+ Memory T cells), CD8+ T cells (Naïve CD8+ T cells, Central Memory CD8 T cells, Effector Memory CD8 T cells, Terminal Effector CD8 T cells), Myeloid dendritic cells, Plasmacytoid dendritic cells	Alpha cells, Beta cells, Gamma cells, Delta cells, Acinar cells, Ductal cells	–	Astrocytes, Endothelial cells, Excitatory neurons, Inhibitory neurons, Microglia, Oligodendrocyte precursors, Neurons, Oligodendrocytes, Excitatory (other neurons), Inhibitory (other neurons)	Proximal tubule, Distal convoluted tubules, Endothelial cells, Collecting duct principal cells, Collecting duct intercalated cells, Loop of Henle, Podocytes, Macrophages, Neutrophils	–
[62]	–	–	Endothelial cells, T cells, B cells, macrophages	–	–	–	–	–

#### 3.2.6 Predominant DL architectures: design, training and optimization pipelines

Most DL-based deconvolution tools employ MLP architectures, accounting for 11 out of the 13 tools analyzed (85%). Only three tools utilize different architectures:Autoptcr implements a CNN with four one-dimensional convolutional layers followed by an MLP with a single hidden layer. TAPE, on the other hand, employs a standard autoencoder consisting of two symmetric components (encoder and decoder), each with four fully connected layers; and CONVdeconv features a novel hybrid architecture.

Regarding the development frameworks used, six tools (46%) were developed in TensorFlow, four (31%) in PyTorch, and the remaining three do not specify their development framework.

MLP-based tools employ different strategies. For instance, Scaden and HASCAD use a framework with three distinct MLPs, each estimating cellular proportions. The final prediction is derived from a weighted average of these estimates. In NNICE, it is introduced the use of a neural network model called Deep Quantile Neural Network (DQNN). Unlike traditional neural networks, which predict only the expected value of an output variable, DQNN estimates multiple quantiles of the output variable distribution, which is particularly useful for understanding uncertainty and possible outcome ranges. NNICE calculates cell proportions individually, using one MLP per target cell type for each defined quantile, specifically analyzing 5 quantiles and 6 cell types. DQNN MLPs differ slightly from the classical model by employing a quantile-specific loss function that penalizes deviations asymmetrically according to the desired quantile.

DeepDecon is an iterative DL model to enhance prediction accuracy. It adjusts predictions based on discrepancies between predicted and actual malignant cell fractions, utilizing 55 MLPs, each with four hidden layers, operating across different malignant cell fraction ranges. Lastly, DISSECT incorporates a dual loss function (Tabla 4), combining a reconstruction loss, which measures the model accuracy in reproducing input data, with a consistency loss that encourages similar results between real and simulated RNA-seq samples.

CONVdeconv introduces a hybrid model that combines multiple architectural components, including a convolutional layer, a two-layer MLP, and an attention block, representing a more advanced approach to cellular deconvolution.

The remaining tools rely on simpler MLP architectures, ordered by increasing complexity. Yan et al., 2023 [58] employed a single hidden layer architecture, followed by GMBPurity with two hidden layers. Both DAISM-DNN and Digitaldlsorter feature three hidden layers, while UCDBase incorporates the most complex architecture with four hidden layers.

**Structure of input and output layers**

All tools process transcriptomic data from both real bulk RNA-seq samples and in silico simulations as numerical vectors, containing as many values as the number of genes found in the sample. Consequently, the number of neurons in the input layer varies depending on the selected tool and, within each tool, on the tissue being deconvolved, as the number of expressed genes differs across tissues. This particularity may necessitate adjustments to the neural network within the same tool, which can complicate its usability and limit its versatility. For example, in Digitaldlsorter method, the transcriptomics vector for BC samples contains expression data for 34145 genes, whereas for CRC samples, the vector includes 23039 genes. Similarly, the number of neurons in the output layer must be adjusted when the deconvolution target changes, as different cell types will be expressed depending on the tissue. For instance, in TAPE, the model considers 7 cell types for brain tissue, 5 for pancreatic tissue, and 12 for PBMCs.

It is important to note that the amount of input data does not always directly correlate with the granularity achieved in deconvolution. This becomes evident when comparing Scaden and HASCAD, both employing similar architectures with three MLP layers. The former uses data from 10000 genes to deconvolute 5 PBMC cell types, extending to a maximum of 7 types in pancreatic and brain tissues. In contrast, the latter achieves deconvolution of up to 15 PBMC cell types with data from only 2371 genes. Notably, GBMPurity, despite using nearly 6000 genes, provides only a single tumor purity estimate.

**Models training**

When it comes to model training, significant differences emerge. Tools such as Digitaldlsorter, Scaden, and TAPE, which are capable of deconvoluting multiple tissue types, determine their final hyperparameters using the grid search technique to optimize performance. For Digitaldlsorter, this process relies solely on data from CRC patients; Scaden uses PBMC and ascites data, while TAPE also works with PBMC data. This targeted hyperparameter tuning approach may restrict model performance when applied to tissues beyond those used for optimization. In contrast, models such as CONVdeconv and GBMPurity focus exclusively on a single tissue type, tailoring hyperparameter selection specifically for that use case.

Furthermore, the availability of training datasets differs across tissues within the same model, leading to inconsistencies in training. For example, methods such as Digitaldlsorter, Scaden, TAPE, and DISSECT operate under different training conditions. The number of training epochs, in particular, differs significantly between approaches: HASCAD is trained for 20 epochs, Digitaldlsorter for 50 epochs, and Scaden for 5000 steps. DISSECT, on the other hand, is trained for 5000*k* epochs, where *k* corresponds to the number of target cell types. Notably, GBMPurity trains the model until convergence, allowing the network to reach an optimized stable state.

In terms of architecture, Table 4 illustrates that most models use the SoftMax activation function in the output layer. This function converts a vector of real numbers into a probability distribution, commonly used in multiclass classification models. It ensures the output probabilities sum to one, with the predicted class corresponding to the highest probability in the output vector. The optimizer used across all models is Adam, with the only variation being the learning rate.

Despite the classification elements in the models, the core task remains regression, as indicated by the employed loss functions. Common regression losses include L1, Mean Squared Error (MSE), Mean Absolute Error (MAE), Root Mean Squared Error (RMSE) and Pearson Correlation Coefficient (r). The only exception is Kullback-Leibler divergence, used by Digitaldlsorter, which measures the difference between two probability distributions and is typically used in classification tasks to assess the divergence between actual and predicted classes.

These considerations are crucial when evaluating the training processes and outcomes of these models. The combination of regression and classification tasks may impact overall performance, a topic that will be discussed further in the next section.

#### 3.2.7 Benchmarking

One key challenge in evaluating DL models for deconvolution lies in comparing their performance against both traditional and neural methods [5]. Most benchmarked tools are non-neural due to their established use and broader prevalence, with CIBERSORTx (CSx) and MuSiC being the most common, appearing in eight of the thirteen reviewed publications. Less frequently used tools include CIBERSORT (CS), quanTIseq, and MCP-Counter [66], found in four and three studies, respectively. In contrast, DL-based tools, though more recent and innovative, remain less numerous. Despite the growing interest in DL models, they are not always benchmarked against other neural-based methods. For instance, Scaden, which is the leading DL method, is only referenced in seven out of the thirteen studies for comparison.

When comparing different deconvolution algorithms, it is important to consider tools requirements. For instance, MCP-Counter relies on specific gene signature matrices, while MuSiC requires scRNA-seq reference data. Neural methods, such as Scaden, need bulk RNA-seq data for network training. Additionally, each tool is designed with specific objectives, which determine the types of tissues they can analyze and their resolution, as discussed in the previous section.

Typically, the first comparisons are conducted using simulated datasets, followed by real datasets. Various metrics are employed to evaluate model accuracy, such as error magnitude (MAE, RMSE, MSE) and correlation (CCC, r) between real and predicted values.

Initially, Digitaldlsorter evaluated its performance through correlation plots comparing its results with those from traditional tools such as TIMER, ESTIMATE, EPIC, and MCP-Counter. At that time, DL-based deconvolution tools had not yet emerged, making these comparisons the standard for evaluation. Rather than focusing on the accuracy of the tool, the evaluation focused on how closely its results aligned with those of other established methods in the field.

Methods such as Autoptcr, HASCAD, NNICE, or the one proposed by Yan et al. [58] do not compare their performance with other deconvolution neural models. Autoptcr paper, for instance, performs its benchmarking against CPM [67], CS, CSx, and MuSiC using four simulated PBMC datasets and one real PBMC dataset, showing the highest correlation and lowest error on simulated data, although all tools exhibited low correlation (<0.5) when deconvoluting the real dataset. HASCAD tool was evaluated against CSx and quanTIseq using nine real PBMC datasets, outperforming CSx and matching quanTIseq performance while also being able to analyze six additional cell types. Meanwhile, Yan et al., 2023 method surpassed an NNLS-based tool in all simulations across various tissues (brain, breast, colon, kidney, liver, and lung), achieving correlation coefficients (r-values) of 0.98 and 0.79, respectively. Additionally, NNICE approach demonstrated superior correlation metrics compared to TIMER [68], quanTIseq, EPIC, MCP-Counter, and CS using both simulated and real PBMC data, showing correlations of 0.7.

The DAISM-DNN method was compared against several deconvolution tools, including Scaden, MuSiC, CS, CSx, ABIS [69], EPIC, quanTIseq, MCP Counter, and xCell, using the real RNA-seq dataset SDY67. It outperformed all methods, achieving the lowest RMSE and highest correlation (r, CCC) across 30 permutation tests, with particular superiority over Scaden. Further analysis was conducted to determine if DAISM-DNN's superior performance was solely attributed to the training data generated by the DAISM method. When both Scaden and DAISM-DNN were trained on simulated PBMC data augmented with SDY67 calibration data, their performances became comparable, with improvements observed in both models.

Additionally, TAPE was benchmarked against Scaden, RNASieve [70], CSx, DWLS [71], MuSiC, and Bisque [72], using simulated bulk data from the Tabula Muris Atlas in three scenarios: normal, rare, and similar. Neural methods were more robust in all three scenarios, but DWLS outperformed them in the normal scenario. On real PBMC and brain datasets, TAPE exhibited the highest MAE values and lowest variance, while Scaden had higher correlation values.

In the development of DeepDecon, the benchmarking was again performed with Scaden, CSx, Bisque, ESTIMATE, MuSiC, MEAD [73], RNA-Sieve, and an NNLS-based model, using datasets from AML,^22^ HNSCC,^23^ and neuroblastoma patients. DeepDecon outperformed its counterparts in 11 of 15 AML datasets and achieved the lowest RMSE and highest correlations for HNSCC and neuroblastoma datasets.

The UCDBase evaluation of RNA-seq deconvolution included SCDC [74], MuSiC, and Scaden. The results showed that the method achieved a mean *r*-value score of 0.68 when deconvolving 96 cell mixtures.

In CONVdeconv, comparisons are made using simulated data with two DL-based deconvolution tools: Scaden and DestVI [75], a spatial transcriptomics deconvolution model. Notably, both Scaden and DestVI exhibit negative *r*-values, whereas CONVdeconv achieves an *r*-value of 0.86. Additionally, CONVdeconv attained the lowest RMSE and MAE values (RMSE = 0.07, MAE = 0.06).

GBMPurity benchmarking is performed against MuSiC, CIBERSORTx, PUREE [76], and Scaden. A comparison across two bulk RNA–seq datasets shows that the PUREE (MAE = 0.102, RMSE = 0.123, *r* = 0.803, CCC = 0.701) and GBMPurity (MAE = 0.128, RMSE = 0.160, *r* = 0.757, CCC = 0.743) models yield the best metrics when estimating tumor purity in glioblastoma within the EORTC and TCGA datasets, respectively. Scaden performs the worst in both cases.

Finally, DISSECT expanded neural tool comparisons to include Scaden, TAPE, and an MLP, as well as traditional methods such as MuSiC, CSx, BayesPrism [73], and b-MIND [77]. Metrics such as JSD (Jensen-Shannon Divergence) were used for additional evaluations, and DISSECT consistently emerged as the top performer across PBMC, brain, pancreas, and kidney datasets, though the second-best tool varied by tissue type: TAPE for kidney, the MLP for brain, and Scaden for PBMC.

In summary, neural-based tools are increasingly featured in comparative deconvolution studies, highlighting their growing importance in the field. In this benchmarking evaluations, DL models generally outperform traditional methods, although their effectiveness varies across tissue types and datasets, emphasizing the need for context-specific assessments.

## 4 Discussion

Deep learning-based deconvolution methods have demonstrated significant potential in bulk RNA-seq analysis. However, our review has highlighted several fundamental challenges that still remain unresolved. It is of particular concern the model design, data preprocessing, and the lack of generalization across different tissues. Addressing these limitations is crucial for ensuring the reliability of these methods in clinical and biomedical research.

**Computational Field.** While this review has a strong focus on DL-based deconvolution methods, classical statistical approaches remain valuable tools in many practical settings. Methods such as linear regression, non-negative matrix factorization (NNMF), or marker-based rule systems can provide accurate results when applied to well-characterized tissues, where cell types exhibit distinct gene expression profiles. These approaches are often easier to implement, computationally less demanding, and more interpretable, advantages that are particularly relevant in resource-limited or time-sensitive contexts.

In contrast, DL models have shown promising results in more challenging scenarios, such as highly heterogeneous tissues, dealing with noisy data, or cases where cell type signatures overlap. Their capacity to learn complex, non-linear relationships make them particularly useful when manual feature selection or prior knowledge is limited. Notably, all the DL-based methods included in this review report benchmarking against traditional approaches, often demonstrating improved performance in terms of accuracy or robustness under such conditions.

Therefore, the decision to use deep learning versus traditional methods should be guided by practical factors, including data complexity, availability of training resources, computational constraints, and the importance of model transparency.

Beyond the choice of the modeling framework, another key challenge in the field is the conceptual framing of the deconvolution problem itself.

One of the most relevant issues highlighted by our review is the ongoing debate on whether cellular deconvolution should be formally categorized as a regression or as a classification problem. Most deep learning-based approaches employ loss functions typical of regression tasks, such as Mean Squared Error (MSE), to estimate cell type proportions. However, a significant number of implementations incorporate a SoftMax function in the output layer. Although SoftMax is more commonly used in classification tasks, its purpose is simply to normalize outputs so they sum to unity, making the suitable for representing compositional data, such as cell type fractions or probabilities. This has led to a methodological duality: while the objective is to estimate continuous proportions (suggestive of regression), the use of SoftMax introduces a probabilistic interpretation often associated with classification. Clarifying this conceptual ambiguity and understanding its impact on model performance is essential. It also opens the door to the possibility that, in certain contexts, a classification-based formulation could be more appropriate, particularly when the goal is to assign dominant cell types or when working with discretized labels.

Another critical aspect is whether the application of a given neural network architecture, trained with data from a specific cell type, to deconvolve different tissues without making modifications to the internal structure of the model is a well suited problem. In this regard, tools like Scaden are optimized using data from PBMCs and then apply the resulting architecture to deconvolve pancreatic or brain tissues. This strategy raises questions about the validity of assuming that gene expression patterns are comparable across these different contexts, given that gene expression and regulatory cell interactions are highly dependent on the considered tissue. This tissue-dependency suggests that the direct transfer of a model optimized within a specific tissue context to analyze the composition of a different one may not be appropriate without a more thorough adjustment. We would like to emphasize the necessity of a robust study on the adaption of each model to the specific characteristics of the tissue under study.

Furthermore, it is noteworthy that even within the same tool, the number of samples used to train the model varies significantly between different tissues. This potential inconsistency could affect the robustness and generalization of the models, suggesting that greater standardization in data usage and training methodology is crucial to enhance the effectiveness of deconvolution methods.

An additional significant challenge arises when we want to compare the effectiveness between different deconvolution models. Each of the revised tools handles data differently and presents varying levels of granularity, leading then to discrepancies in both their inputs (genes) and outputs (cellular proportions or classes). Furthermore, the existence of diverse validation methods complicates this comparison, as there is no single standard criterion for evaluating and comparing the results obtained by each model. This lack of homogeneity sticks out the need of establishing a uniform set of criteria and protocols in the field of cellular deconvolution.

Finally, although most current models are based on the same architecture as Scaden, i.e., an MLP network, recent efforts are devoted to implement more sophisticated models, such as Autoptcr or TAPE, which are based on CNNs and Autoencoders, respectively. This recent evolution indicates that the field is still in its infancy. This straightforward reliance on simple models leaves room for significant improvement. Future work in this field could focus on implementing more advanced architectures, such as attention-based models or graph neural networks, which may be better suited to capture the rich variability inherent in transcriptomic data. Another rather weak point for deconvolution tools training is the scarcity of real data. A great amount of effort has been devoted lately to build reliable generators of synthetic scRNA-seq data, so more realistic pseudobulks can be implemented to make more robust tools [78], [79], [80].

**Biological Field.** In the biological realm, as we have already mentioned above, the scarcity of RNA-seq data represents a significant obstacle for constructing robust models. Currently, there is a reliance on generating *in silico* data that attempts to simulate RNA-seq data using scRNA-seq data. However, intrinsic differences between these data types, which are not yet fully understood, could affect the proper training of the models. Additionally, the process of generating these pseudobulks varies considerably between studies, as we have noted in this review, highlighting the lack of standardization in this aspect.

Moreover, access to annotated scRNA-seq data, necessary for generating training pseudobulks, presents an additional challenge. Often, these data are unlabeled, requiring a labor-intensive, complex process that may be difficult to reproduce without the original pipeline. Therefore, it is essential to develop a consensus on best practices for the generation and preprocessing of transcriptomics data, as this could improve the consistency and comparability of results obtained with deep learning models.

Regarding the studied tissues in the analyzed manuscripts, it is notable that most samples used for training and evaluation come from Peripheral Blood Mononuclear Cells (PBMCs). This predominance is largely due to the accessibility and well-characterized nature of PBMCs, making them a common benchmark dataset in the field. However, it is important to clarify that most deconvolution tools are not inherently limited to PBMC data and can be generalized to other tissues provided that appropriate training data or reference profiles are available. Nonetheless, the predominance of PBMC-based studies raise questions about the applicability and robustness of these tools across a wider variety of tissue types and clinical contexts. Only one of the articles included in our review specifically addresses deconvolution in two distinct types of cancer, highlighting the still limited scope of these methods beyond blood-derived samples. It is crucial to develop models capable of deconvolving a wide variety of tissues, rather than being limited solely to PBMC samples, as has been the predominant approach thus far. Expanding the spectrum of tissues addressed could have a significant impact on clinical practice, facilitating the study of various diseases and allowing for a more comprehensive analysis of how cellular proportions evolve in patient samples over time.

Future directions in this field include the need to achieve a representation of RNA-seq data that provides greater insight into the dynamics and interdependence of cells. Cellular states play a crucial role in the variability of gene expression, and the emergence of one cell type can influence the expression of other types. Understanding these interconnections is essential to enhance the accuracy of deconvolution models.

Lastly, it is important to emphasize the need for standardization in data acquisition for training and validation methods, which could clarify the utility of deconvolution models in biomedical research.

**Method Selection.** Selecting the most appropriate deconvolution method should be guided by practical factors such as data characteristics, resource availability, and research objectives. For datasets derived from well-characterized tissues where cell types are separated by distinct marker genes, classical methods—such as non-negative least squares (NNLS), linear regression, or rule-based approaches—often provide sufficient accuracy with the added benefits of speed, simplicity, and interpretability. When predefined signature matrices are unavailable or unreliable, hybrid tools like CIBERSORTx, MuSiC, or CDSeq offer effective alternatives, as they can infer cell type signatures directly from bulk or single-cell data. For more complex biological systems, such as heterogeneous tumors with overlapping expression profiles or extensive cellular plasticity, deep learning-based models like Scaden, TAPE, or DeepDecon offer superior performance due to their ability to capture non-linear relationships and generalize across tissue types.

However, deep learning methods come with trade-offs, including higher computational demands, increased complexity, and reduced interpretability. Therefore, for studies with limited computational resources or where transparency is crucial (e.g., clinical diagnostics), traditional or hybrid tools may be preferred. In some cases, a combined approach may be useful, for instance, applying classical tools for initial screening followed by deep learning-based refinement. Additionally, when inter-individual variability is of interest, tools that allow dynamic or personalized signature generation (e.g., CIBERSORTx, MuSiC, or VAE-based models) provide greater flexibility.

Ultimately, no single tool is universally optimal. Method selection should take into account not only performance metrics but also the biological context, technical variability, and interpretability needs of the study. We encourage users to benchmark tools using validation datasets that match their specific conditions and to consider ensemble strategies when appropriate.

## 5 Conclusions

In this review we have examined several deep learning-based cell deconvolution tools, highlighting their key methodological and conceptual challenges, and hinting to the potential future research directions in this rapidly evolving field.

A major common issue we have encountered through this revision is the wide variability in the outputs generated by each tool, including not only the differences in the selected cell types that are identified but also in the format of their results. This heterogeneity rather complicates any direct comparison between different methods, therefore emphasizing the necessity of standardized evaluation criteria to enable an objective benchmarking.

There is an ongoing debate concerning the best approach to tool development. While some studies aim to create generalized models that can adapt to samples coming from different tissues and obtained in diverse experimental conditions, others studies sustain that focusing on specialized models optimized for specific applications is better suited to the variable nature of transcriptomics data. Generalized models offer the advantage of broad applicability, as they can be used across multiple datasets and conditions without requiring major architecture changes. This flexibility is particularly useful in large-scale studies where samples come from different sources, as well as in clinical settings where robustness across various patient-derived samples is crucial. However, this generalizability often comes at the cost of decreased precision, as these models may not fully capture tissue-specific transcriptomics signatures.

On the other hand, specialized models tailored to specific tissues or experimental settings can achieve higher performance by leveraging domain-specific features and optimizing hyperparameters accordingly. These models are particularly beneficial in applications where fine-grained cellular resolution is needed, such as tumor microenvironment studies or immune profiling in disease contexts. Nevertheless, their applicability is more limited, as they may require retraining or fine-tuning when applied to different datasets, reducing their usability in diverse research scenarios.

Both strategies present different compromises, and their selection depends on the intended application, the availability of training data, and the desired balance between accuracy and generalizability. Future developments in this field may involve hybrid approaches that integrate the strengths of both methodologies, potentially leveraging meta-learning or transfer learning techniques to enhance adaptability without sacrificing precision.

Moreover, almost all of the developed deconvolution models rely on relatively simple deep learning architectures. Henceforth there is still significant room for improvement through the implementation of more sophisticated models capable of better capturing the complexity of transcriptomics data. Additionally, transcriptomics data has traditionally been represented as vectors, yet alternative representations, such as image-based formats or structured data models, could enhance the predictive power of these alternative models and provide novel insights that can potentially be more accurate in the deconvolution tasks.

Finally, the generation of synthetic data tailored to the specific challenges of cell deconvolution emerges as a potential key strategy for improving model training and validation. The development of well-characterized datasets that accurately reflect the biological heterogeneity of tissues would enhance model robustness and broaden their applicability in both research and clinical settings.

## Funding statement

This study has been funded by 10.13039/501100004587Instituto de Salud Carlos III (ISCIII) through the project PI22/00492 and cofunded by the 10.13039/501100000780European Union (FIS PI22/00492) and Ayudas a la Investigación 10.13039/100012986UFV Grant (UFV2023-24).

## CRediT authorship contribution statement

**Alba Lomas Redondo:** Writing – original draft, Formal analysis, Data curation. **Jose M. Sánchez Velázquez:** Writing – review & editing, Writing – original draft, Supervision. **Álvaro J. García Tejedor:** Writing – original draft, Supervision, Conceptualization. **Víctor Javier Sánchez–Arévalo Lobo:** Writing – review & editing, Writing – original draft, Supervision, Funding acquisition, Conceptualization.

## Declaration of Competing Interest

The authors declare that they have no known competing financial interests or personal relationships that could have appeared to influence the work reported in this paper.

## Footnotes

1 https://www.ncbi.nlm.nih.gov/geo/.

2 https://www.10xgenomics.com/.

3 https://immport.org/.

4 https://www.cancer.gov/ccg/research/genome-sequencing/tcga.

5 https://zenodo.org/.

6 http://tisch.comp-genomics.org/home/.

7 https://singlecell.broadinstitute.org/single_cell.

8 https://atlas.brain-map.org/.

9 https://www.cbioportal.org/.

10 https://portal.gdc.cancer.gov/.

11 http://linkedomics.org/.

12 https://www.gtexportal.org/home/.

13 https://www.cgga.org.cn/.

14 https://ega-archive.org/.

15 https://cellxgene.cziscience.com/datasets.

16 http://xteam.xbio.top/CellMarker/.

17 https://github.com/SydneyBioX/scClassify.

18 https://github.com/jdekanter/CHETAH.

19 https://bioconductor.org/packages/zinbwave/.

20 https://scanpy.readthedocs.io/.

21 https://satijalab.org/seurat/.

22 Acute Myeloid Leukemia.

23 Head and Neck Squamous Cell Carcinoma.
